# Characterization and genetic diversity of pseudomonads population from highbush blueberry in western Canada

**DOI:** 10.1007/s00253-025-13676-y

**Published:** 2026-01-12

**Authors:** Someshwar R. Latchman, Rishi R. Burlakoti, Amy Novinscak, Simone D. Castellarin

**Affiliations:** 1 Agassiz Research and Development Centre, Agriculture and Agri-Food Canada, 6947 Hwy 7, Agassiz, BC V0M 1A0 Canada; 2 Wine Research Centre, Faculty of Land and Food Systems, The University of British Columbia, Vancouver, BC Canada

**Keywords:** *Pseudomonas syringae*, Blueberry, Rep-PCR, Housekeeping genes

## Abstract

**Abstract:**

Bacterial blight (causal agent *Pseudomonas syringae* complex, Psc) is an endemic and economically important disease of northern highbush blueberry production in Canada and the Pacific Northwest of the USA. To date, there is no comprehensive survey of the disease in the region and detailed characterization of associated pathogens from Pacific western Canada. Therefore, we did comprehensive disease survey and characterization of associated pseudomonads population using pathogen morphology, biochemical tests, and molecular characterization. We isolated 380 strains of pseudomonads from symptomatic plants from 32 research and commercial fields in 10 diverse geographic locations in British Columbia. We used *P*. *syringae* specific (Psy) primers and identified 197 Psy-PCR positive isolates out of 380. We further sequenced Psy-PCR positive isolates of pseudomonads using four housekeeping genes and identified four phylogenomic species: *P. syringae* (40%), *Pseudomonas avellanae* (29%), *Pseudomonas viridiflava* (20%), and phylogenomic species A (7%). *P*. *avellanae* and *P. viridiflava* are new phylogenomic species of Psc causing bacterial blight in highbush blueberry. We found some patterns among geographical locations and highbush blueberry varieties in the frequency distribution of isolates of these phylogenomic species. Genetic fingerprinting with rep-PCR assays identified a very high genetic diversity of pseudomonads populations among geographical locations, varieties, and phylogenomic species. Biochemical characterization (LOPAT- levan, oxidase, pectolytic activity, arginine dihydrolase, and tobacco hypersensitivity) revealed that the vast majority of isolates were *Pseudomonas* Group Ia. Findings of this study provide insight into the population biology of pseudomonads infecting highbush blueberry, provide information for disease diagnosis, and exploit disease management options, including identifying sources of disease resistance.

**Key points:**

• *High prevalence of bacterial blight caused by P. syringae complex (Psc) in highbush blueberry in Pacific western Canada*

• *We report two new phylogenomic species of Psc, P. viridiflava and P. avellanae, that cause bacterial blight and canker disease in highbush blueberry*

• *The genetic diversity of the population of Psc was very high*

**Supplementary information:**

The online version contains supplementary material available at 10.1007/s00253-025-13676-y.

## Introduction

Blueberry is a valuable global fruit crop. In Canada, blueberry (highbush and lowbush) ranked as the largest fruit crop by production areas and the second largest by farm gate values in 2023 (Agriculture and Agri-food Canada 2024). Highbush blueberry (*Vaccinium corymbosum*) contributed $170 million in farm gate value in Canada in 2023. British Columbia (BC) is the largest producer of highbush blueberry in Canada, and accounts for 90% of farm gate value and 92% of production area in 2023. Several fruit, cane, foliar, and root diseases caused by fungi, oomycetes, bacteria, and viruses are concerns for the highbush blueberry growers in BC, Pacific western Canada (Cline 2014; Martin et al. 2012; Anonymous 2025).

Bacterial blight and canker caused by the bacterium *Pseudomonas syringae* complex (Psc) is an endemic disease of highbush blueberry in the western area of Canada and the Pacific Northwest region of the United States (BC, Washington, and Oregon). The pathogen causes damage to young blueberry plants by infecting floral buds and causing canker and dieback symptoms on apical stems; however, bacterial blight symptoms also occur on leaves and fruit appearing as spots and blisters (Moore 1988; Scheck et al. 1997). Bacterial blight significantly impacts newly planted blueberry orchards and also causes significant reduction in fruit yields of susceptible varieties of highbush blueberry, as the pathogen damages the floral bud. The pathogen enters into plant tissues through wounds, injuries caused by mechanical damage or insects, or natural openings of the host tissues. The pathogen survives on infected tissues of highbush blueberry, and also overwinters on orchard groundcovers, leaf litter, and weed hosts (Hirano and Upper 1983; Monteil et al. 2012; Lamichhane et al. 2015). The pathogen can grow and survive on asymptomatic host tissue epiphytically and endophytically throughout the entire growing season. Due to the epiphytic nature of *P. syringae* isolates, pathogen populations decrease considerably in the summer, especially if temperatures exceed 25 °C (Scortichini 2019). The epiphytic populations of *P. syringae* can increase during the autumn season when temperatures decrease and the frequency of rainfall increases (Morris et al. 2013). *P*. *syringae* can also survive and multiply at freezing temperatures, as well as produce membrane-bound proteins that nucleate ice to cause freezing damage to host plants (Hirano and Upper 2000). Ice nucleation results in the release of nutrients through host cell damage, thus facilitating growth and spread of *P. syringae* (Hirano and Upper 2000).

Several microbial and biochemical methods are used for the phenotypic characterization of pseudomonads strains (King et al. 1954; Mohan and Schaad 1987; Braun-Kiewnick and Sands 2001). Microbiological methods include growing bacterial isolates in semi-selective (King’s B) medium and observing colony morphology and fluorescence under UV light. The LOPAT (levan, oxidase, pectolytic activity, arginine dihydrolase, and tobacco hypersensitivity) assay is the most common approach for the biochemical characterization of pseudomonads (Lelliott et al. 1966; Latorre and Jones 1979; Hamedan and Harighi 2014; Keshtkar et al. 2016; Gerin et al. 2019). Molecular characterization of pseudomonads strains includes species-specific polymerase chain reaction (PCR) assays (Guilbaud et al. 2016), multilocus sequencing with housekeeping genes, and genetic fingerprinting with repetitive element-based PCR assays (rep-PCR) (Abdellatif et al. 2017; Gomila et al. 2017; Nikolić et al. 2018).

The occurrence of bacterial blight in highbush blueberry in BC was previously documented, and the causal agent was considered to be *P*. *syringae* pv. *syringae* (Canfield et al. 1986; MacDonald 1990; MacDonald et al. 2002; Joshi 2003). The previous nomenclature of the pathogen at the pathovar level was primarily based on hosts from which pseudomonads were isolated, biochemical characterization, and pathogenicity assays; these traditional methods are not sufficient to delineate at species or phylogenomic species levels (Young, 2010). With the limited control measures available to the highbush blueberry growers, bacterial blight and canker are still widespread in BC and occur yearly with moderate to high disease pressure (Anonymous 2025). In neighboring states of the USA (Washington and Oregon), the disease is also reported as a major sporadic problem for highbush blueberry (Scheck et al. 1996; Strik 2006). Characterization of pathogen strains is a component of pre-breeding activities for developing disease-resistant host genotypes and applying other management options to control the disease. To date, there is no comprehensive survey of the bacterial blight disease from diverse fields representing multiple varieties in BC. In addition, there is a lack of scientific studies for phenotypic and genetic characterization of a large population of pseudomonads associated with the disease. Therefore, the objectives of this study were to (i) assess the prevalence of bacterial blight and canker in highbush blueberry of diverse varieties across growing regions in BC; (ii) identify and characterize the large collections of pseudomonads strains isolated from diverse geography and varieties of highbush blueberry; and (iii) examine the phylogenomic species composition and genetic diversity of the pseudomonads populations among diverse geography and varieties of highbush blueberry.

## Materials and methods

### Disease monitoring, sample collection, and bacterial isolation

**Fig. 1:**
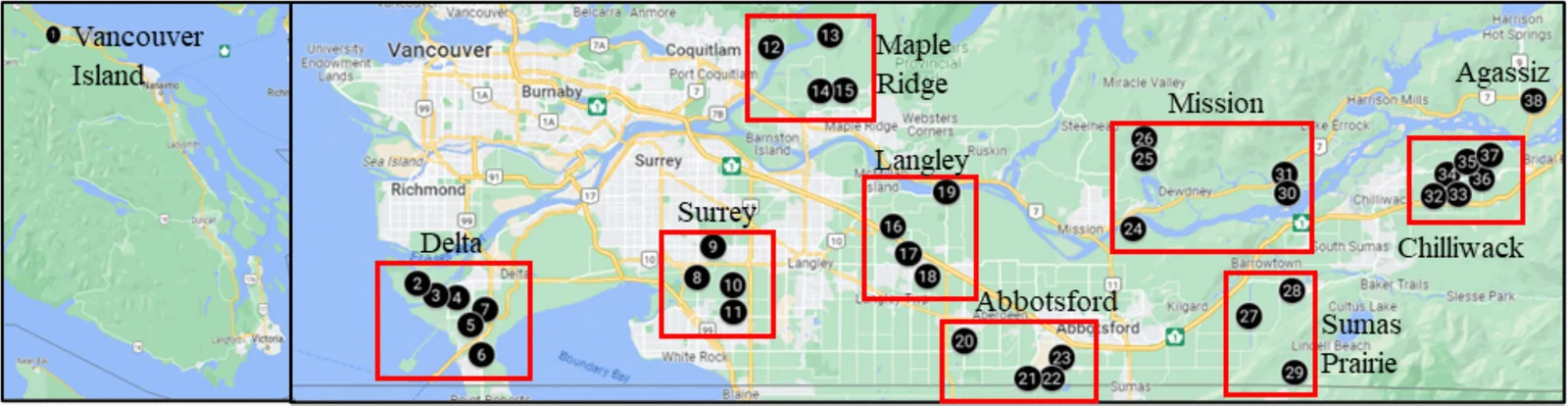
Map showing the field sites (numbers in black circles) monitored for the prevalence of bacterial blight and sampling of diseased tissues of highbush blueberry

Highbush blueberry fields from research plots and commercial farms (*n* = 38) of nine regions of the Lower Mainland region (Delta, Surrey, Maple Ridge Langley, Abbotsford, Mission, Sumas Prairie, Chilliwack, and Agassiz) and near Qualicum Beach of Vancouver Island in BC were monitored for bacterial blight, and symptomatic plant samples (e.g., stems with floral buds, leaves) were collected between the first week of April and mid-August from 2017 to 2020 (Table 1 and Fig. 1). Fifteen commercial varieties were assessed in the study, which included early-, mid-, and late-season varieties (Table 1). Among the varieties assessed, Duke and Northland were early season; Draper, Reka, and Bluejay were early-mid season; Bluecrop, Liberty, Hardy Blue, and Top Shelf were mid-season; and Aurora, Calypso, Cargo, Chandler, Elliot, and Lastcall were late season varieties. In each field, representative areas of each variety (five to ten rows, depending on the size of the field plots) were monitored for bacterial blight-like symptoms, and plant samples with necrotic twigs were collected. The infected plant materials were sealed in plastic bags and stored at 4ºC prior to isolating the presumptive causal agent. The percentage of diseased fields in each location was calculated as: 100*(the number of symptomatic fields/total number of fields monitored in that location).

**Table 1:** The number of bacterial blight infected fields and varieties of highbush blueberry and pseudomonads positive samples in British Columbia (BC) in 2017, 2018, 2019 and 2020. Brackets include percentages from the total number of samples or from the number of fluorescent isolates on King’s B media (KB) and supplemented King’s B media (KBC)

Location	Year	No. of fields	% diseased fields	Variety	Numbers of				
					Diseased samples	Pseudomonads positive samples	Fluorescent isolates on KB	Fluorescent isolates on KBC	Psy + isolate
Agassiz	2017	1	60	Breeding Materials^a^	12	4	9	8	7
	2020	1	100	Breeding Materials	3	1	3	3	1
Subtotal					**15**	**5 (33)**	**12**	**11 (92)**	**8 (67)**
Chilliwack	2017	1	100	Reka	4	1	4	4	3
	2018	1	100	Unknown	12	1	12	12	1
	2019	6	50	Bluecrop	2	1	3	1	3
				Draper	6	2	17	9	6
				Duke	10	1	1	1	1
				Elliott	2	0	5	1	0
				Reka	2	0	2	0	0
Subtotal					**38**	**6 (18)**	**44**	**28 (63)**	**14 (32)**
Mission	2018	1	100	Bluecrop	10	1	10	10	9
	2019	5	40	Bluecrop	3	0	0	0	0
				Chandler	2	0	1	0	0
				Duke	10	2	8	1	2
				Elliott	1	0	0	0	0
				Reka	**1**	0	0	0	0
				Unknown	1	0	3	1	0
	2020	1	100	Duke	2	1	6	6	1
Subtotal					**30**	**4 (13)**	**28**	**18 (64)**	**12 (43)**
Sumas Prairie	2019	3	100	Aurora	5	2	8	7	7
				Duke	4	0	4	0	0
				Cargo	1	0	1	1	0
				Lastcall	1	1	1	0	1
				Reka	1	0	0	0	0
				Top Shelf	1	0	0	0	0
	2020	1	100	Lastcall	2	1	5	5	3
Subtotal					**15**	**4 (27)**	**19**	**13 (68)**	**11 (58)**
Abbotsford	2017	3	33	Unknown	16	3	7	7	4
	2019	4	100	Aurora	5	2	5	4	4
				Bluecrop	1	1	2	2	1
				Draper	6	2	4	4	3
				Duke	6	1	5	2	2
				Elliott	1	1	3	2	2
				Liberty	2	1	2	1	1
Subtotal					**37**	**11 (30)**	**28**	**22 (79)**	**17 (61)**
Langley	2017	1	100	Unknown	3	0	3	0	0
	2018	1	100	Unknown	7	0	7	0	0
	2019	2	100	Bluecrop	2	0	4	2	0
				Chandler	2	1	4	3	1
				Draper	3	1	6	2	2
				Elliott	1	0	0	0	0
				Liberty	3	1	4	1	1
				Last Call	1	0	0	0	0
Subtotal					**22**	**3 (14)**	**28**	**8 (29)**	**4 (14)**
Maple Ridge	2017	2	50	Unknown	5	1	3	1	1
	2019	3	67	Draper	4	1	8	5	1
				Liberty	3	0	0	0	0
	2020	1	100	Bluecrop	1	0	4	4	0
Subtotal					**13**	**2 (15)**	**15**	**10 (67)**	**2 (13)**
Surrey	2019	4	100	Aurora	7	2	12	8	6
				Bluecrop	8	4	11	8	8
				Chandler	2	1	2	2	2
				Draper	2	2	3	3	3
				Duke	1	0	0	0	0
				Elliott	8	3	9	9	9
				Liberty	2	2	3	3	3
				Reka	7	2	4	2	2
	2020	2	100	Aurora	5	5	23	23	16
				Calypso	2	0	14	14	0
				Draper	2	2	6	6	6
				Reka	2	2	5	5	5
Subtotal					**48**	**25 (52)**	**92**	**83 (90)**	**60 (65)**
Delta	2017	1	100	Draper	1	1	3	3	3
				Duke	1	0	1	0	0
				Reka	1	0	2	0	0
	2019	4	100	Bluejay	3	2	5	4	4
				Draper	5	5	12	9	11
				Duke	6	3	16	7	4
				Hardy Blue	10	1	8	1	1
				Northland	3	1	5	1	1
				Reka	6	5	13	8	8
	2020	2	100	Unknown	1	1	3	3	3
				Draper	1	1	2	2	2
				Duke	3	2	8	8	4
				Hardy Blue	3	3	9	9	7
				Northland	1	1	4	4	2
				Reka	1	0	4	4	0
Subtotal					**46**	**26 (57)**	**95**	**63 (66)**	**50 (53)**
Vancouver Island	2018	1	100	Draper	**19**	**4 (21)**	**19**	**19 (100)**	**19 (100)**
Total		52^b^	88		**283**	**90 (32)**	**380**	**275 (72)**	**197 (52)**

^a^ Breeding materials were non-commercial varieties used for propagating new blueberry varieties

^b^ Same fields were visited over different years. There were a total of 38 fields over 10 different locations (Fig. 1)

To isolate the causal agent, four to six pieces of necrotic tissue were selected from each sample bag, surface sterilized with 0.5% sodium hypochlorite (NaOCl) for 45 s and rinsed three times with sterile water. Thereafter, the tissue samples were chopped into small pieces and placed in a sterile Petri dish, and 1 mL of sterile distilled water was added. After 30 min, the suspension was streaked onto King’s B (KB) medium. Single colonies of bacteria were picked after 48 to 72 h and hand transferred to KB medium again. Purified single colony isolates were kept in a 2 mL cryogenic tube with enrichment buffer (20% glycerol, 1% peptone, 0.04% KH_2_PO_4_, 0.1% K_2_HPO_4_) at − 80 °C for long-term storage of Plant Pathology Laboratory in Agassiz Research Centre of Agriculture and Agri-Food Canada, which is a public research institute in Canada. Colonies of all bacterial isolates were further characterized by growth on KB medium supplemented with boric acid, cephalexin, and cycloheximide (KBC) for 48 h. KBC is considered as a semi-selective medium for certain pathovars of *P*. *syringae* including pv*. syringae*, because these pathovars produce a green fluorescent color on KBC when visualized under UV light (Braun-Kiewnick and Sands 2001).

### Genomic DNA extraction of bacterial isolates

DNA extraction was done using Puregene® Core Kit B (Qiagen, Toronto, ON, Canada) for Gram-negative bacteria following the manufacturer’s protocols. The DNA quantity was measured using the Qubit 2.0 (Invitrogen™, Carlsbad, CA, USA), while the quality was determined using the Biotek Synergy HTX multi-mode reader (Agilent, Santa Clara, CA, USA).

### Identification of bacterial isolates with *P. syringae* specific markers

PCR primers specific to *P. syringae* developed by Guilbaud et al. (2016) (Table 2) were used to determine if the bacterial isolates from diseased tissues were *P. syringae*. These primers yield a 144 bp amplicon in *P. syringae* isolates. Each PCR reaction mixture (total volume of 25 μL) contained 12.25 μL of sterile water, 5.0 μL of 5× GoTaq PCR buffer (Promega, Charbonnières-les-Bains, France), 0.5 μL of dNTP mix (10 mM each), 2.5 μL of each primer (5 μM), 0.15 μL of GoTaq DNA polymerase (5 units μL^−1^), and 2.0 μL of DNA template (40 ng μL^−1^). All PCR reagents were purchased from New England Biolabs. PCR amplification was conducted in a Bio-Rad (Mississauga, ON, Canada) thermal cycler. The amplification cycles were 5 min at 96 °C; 30 cycles of 30 s at 94 °C, 30 s at 61 °C, and 30 s at 72 °C; and a 10 min final extension at 72 °C. Amplified PCR fragments were subjected to electrophoresis in 1.5% agarose gels (w/v) with 1× Tris-acetate-EDTA (TAE) buffer and SYBR safe diluted 1:10,000 (Thermo Fisher Scientific, Ottawa, Canada). The gels were visualized using a GelDoc XR+ (Bio-Rad, Mississauga, Canada), and the fragments were estimated using a 100 bp DNA ladder (Invitrogen, Mississauga, Ontario, Canada).

**Table 2:** PCR primers for Psy, fingerprinting primers (BOX, and ERIC), and housekeeping (*cts*, *rpoD, gapA,* and *gyrB*) primers

Targeted gene/repetitive element^a^	Primers^b^	Nucleotide sequences (5′ → 3′)	Annealing temp. (°C)	Amplicon size (bp)	Reference
Psy (P)	Psy-F	ATGATCGGA GCGGACAAG	61		Guilbaud et al. (2016)
	Psy-R	GCTCTTGAG GCAAGCACT			
BOX elements (P)	BOXA1R	CTACGGCAA GGCGACGCT GACG	50	−	Clark et al. (1998)
ERIC elements (P)	ERIC2F	AAGTAAGTG ACTGGGGTG AGCG	50	−	Clark et al. (1998)
	ERIC1R	ATGTAAGCT CCTGGGGAT TCAC		−	
*cts* (P,S)	cts-Fp	AGTTGATCA TCGAGGGCG CWGCC	62	650	Sarkar and Guttman (2004)
	cts-Rp	TGATCGGTT TGATCTCGC ACGG			
*rpoD* (P,S)	PsEG30F	ATYGAA ATCGCC AARCG	57	736	Mulet et al. (2009)
	PsEG790R	CGGTTG ATKTCC TTGA			
*gapA* (P,S)	gapA-F	CGCCATYCGCAACCCG	62	690	Sarkar and Guttman (2004)
	gapA-R	CCCAYTCGTTGTCGTACCA			
*gyrB* (P)	UP1E-F	AYGSNGGNGGNARTTYRA	55	875	Yamamoto et al. (2000)
	APrU-R	GCNGGRTCYTTYTCYTGRCA			
*gyrB* (S)	M13(20)-F	TGTAAACGACGGCCAGT			Yamamoto et al. (2000)
	M13-R	CAGGAAACAGCTATGACC			

^a^Primers were used for PCR amplification (P), or sequencing (S)

^b^*F* forward, *R* reverse

### Pathogenicity test

**Fig. 2:**
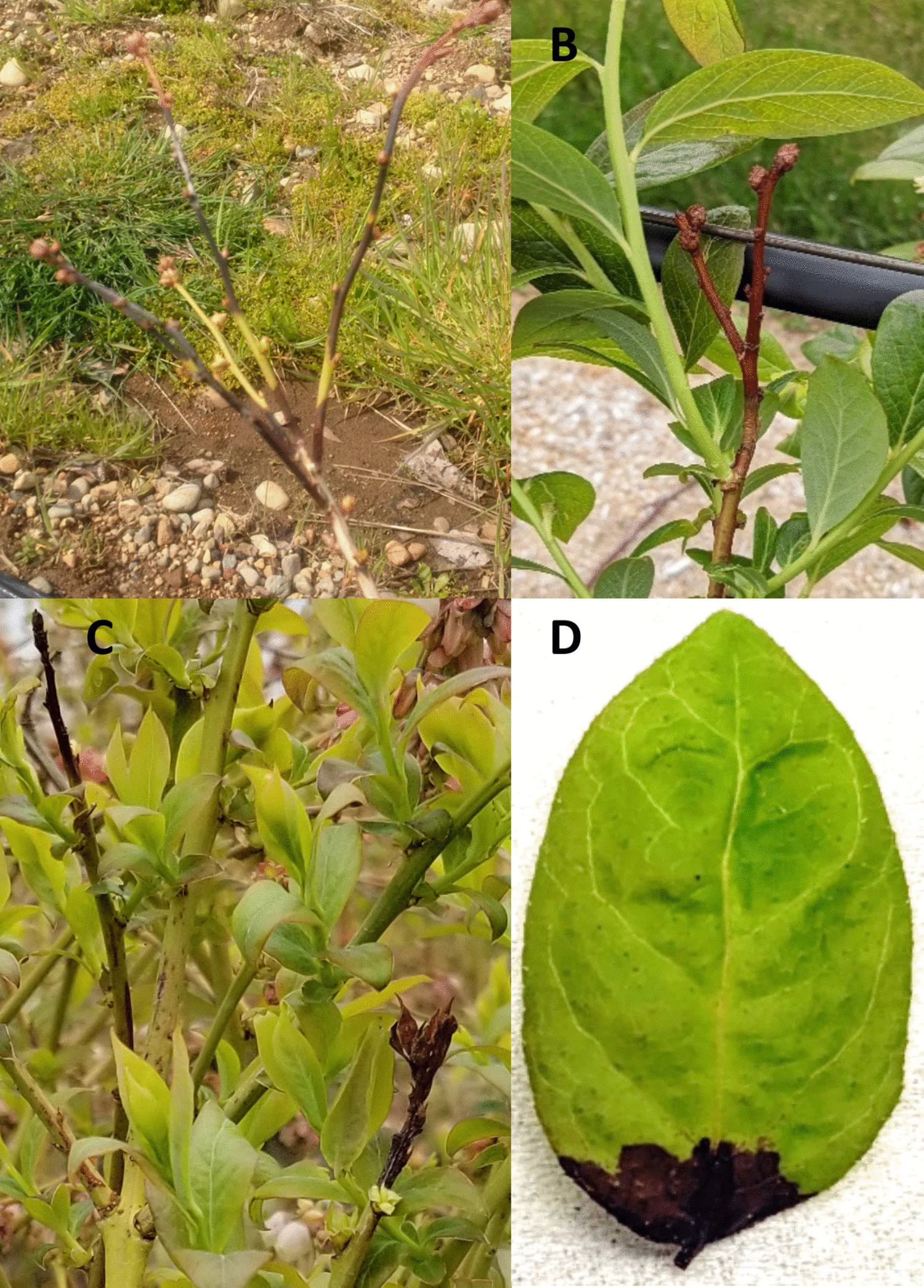
Bacterial blight disease symptoms on highbush blueberry plants in fields (**A**–**C**). **A** Severe blight (black canker) on multiple stems progressing from the tip to the base of stems in young blueberry plants, **B** brown necrosis on floral buds and blight on stems on young plants, **C** black canker on multiple stems, and blight on young leaves of mature blueberry plants. **D** Black necrosis symptoms on detached leaf in pathogenicity test

To confirm the ability of the bacterial isolates to cause disease, bacterial isolates (*n* = 50) from our study and three reference isolates obtained from Dr. Tambong’s laboratory at the Ottawa Research and Development Centre, Agriculture and Agri-Food Canada were inoculated on detached young and healthy leaves of the Draper variety of the highbush blueberry, which is susceptible to bacterial blight. For each isolate, 3 leaves were inoculated in each experiment and repeated independently. Experiments were laid out in a completely randomized design. The blueberry leaves were surface sterilized in 0.5% NaOCl for 10 s then rinsed for 20 s in sterile distilled water three times. The leaves were dried on sterile paper towel in a laminar flow hood. The leaves were wounded with a sterile scalpel at the base of the leaf and inoculated with 25 µL of an overnight bacterial suspension adjusted to an OD of 0.3 (~1 × 10^8^ colony forming unit, CFU mL^−1^). Sterile distilled water was used to inoculate wounded leaves as the control. Three leaves were placed per isolate in the same humidity chamber. Humidity chambers were Petri dishes containing soaked sterile filter papers and wrapped with Parafilm (Bemis, Neenah, USA). Humidity chambers were placed at 4 °C for 24 h to simulate frost damage and then the chambers were transferred to a growth chamber set at 20 °C (16:8 light/dark). The isolates were categorized as non-pathogenic if the lesion length and width were ≤ 2 mm, or pathogenic if the lesion length and width were ≥ 2 mm (Fig. 2D).

### Biochemical characterization

**Fig. 3:**
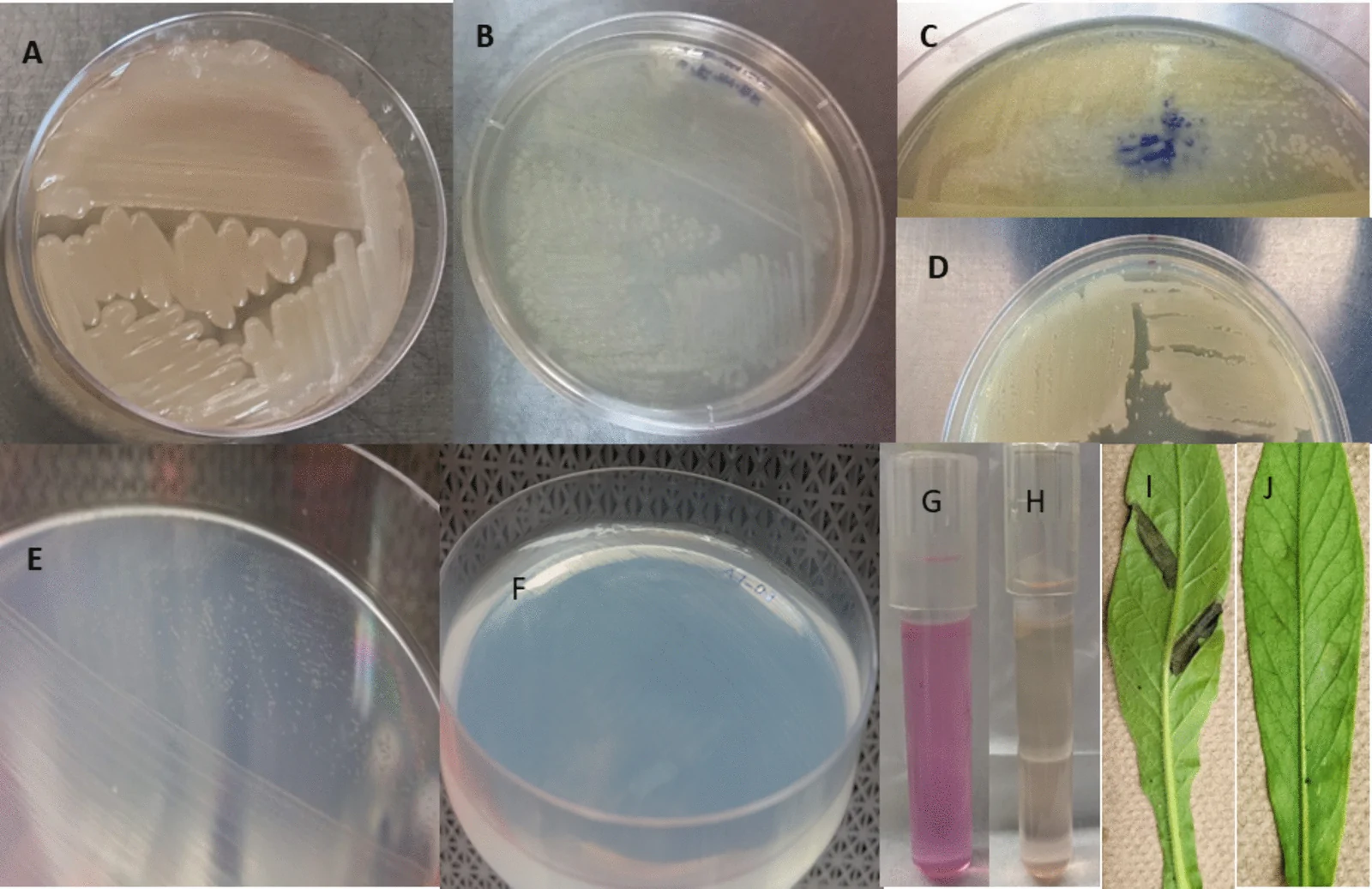
Figures showing positive and negative results of biochemical tests used to characterize the strains of *P. syringae* complex. Positive—white, mucoid, dome-shaped colonies (**A**) and negative—any non-white, non-mucoid, non-dome shaped colonies (**B**) results of levan test. Positive- development of a purple color within 10 s (**C**) and negative—no color development after 60 s (**D**) results of oxidase test. Positive- depression formed in the medium (**E**) and negative- no pectolytic activity/depression in the medium (**F**) results of pectolytic activity test. Positive- color change to deep pink or red (**G**) and negative- no color change (**H**) results of arginine dihydrolase activity test. Positive—necrotic lesion formation along the infiltration site (**I**) and negative—healthy leaves after infiltration (**J**) results of tobacco hypersensitivity test

Biochemical characterization of bacterial isolates (*n* = 100) was further carried out using LOPAT tests, verifying the production of levan (L), oxidase activity (O), pectolytic activity (P), arginine utilization (A), tobacco hypersensitivity (T) by following the procedures outlined by Lelliott et al. (1966) and Braun-Kiewnick and Sands (2001). For each isolate, two independent experiments were conducted with two replicates in each experiment. For the levan production test, bacterial isolates were streaked onto nutrient agar supplemented with 5% (w/v) sucrose and incubated for 3–5 days. A positive test resulted in white, mucoid, dome-shaped colonies (Fig. 3A), and a negative test was any other phenotype (Fig. 3B). For the Kovacs oxidase test (Kovacs 1956), isolates grown on KB medium for 48 h were transferred using a toothpick onto filter paper soaked with 0.1% aqueous solution of *N*, *N*, *N′*, *N′*-tetramethyl-*p*-phenylenediamine dihydrochloride. A positive result was indicated by the development of a purple color within 10 s, and a delayed positive resulted if the color developed within 10–60 s (Fig. 3C). A negative result resulted from no color development after 60 s (Fig. 3D). For pectolytic activity, crystal violet pectate medium (HIMEDIA, Kennett Square, PA, USA) was made according to the manufacturer’s specifications, and *P. syringae* isolates were streaked on the medium. A depression formed in the medium around the growth of the isolate after 24 h at 22 °C indicated a positive result (Fig. 3E), and a negative result showed no pectolytic activity/depression in the medium (Fig. 3F). For arginine dihydrolase activity, Thonley’s medium 2 A (Braun-Kiewnick and Sands 2001) was made and the pH was adjusted to 7.2. Bacterial isolates were streaked on the medium and covered with 1 cm of sterile mineral oil. A positive result is revealed by a color change to deep pink or red within four days (Fig. 3G), whereas a negative result is indicated by no color change in the medium (Fig. 3H). For the tobacco hypersensitivity reaction, a bacterial suspension of ~ 10^8^ colony forming unit per mL (CFU mL^−1^) prepared from an overnight grown culture with an OD (optical density) of 0.3 at wavelength 600 nm, equivalent to ~ 10^8^ CFU mL^−1^, was infiltrated into a tobacco leaf using a syringe. Necrotic lesion formation along the infiltration site of the tissue after 24 h was a positive result (Fig. 3I), whereas healthy leaves indicated a negative result (Fig. 3J).

### Multilocus sequencing with amplification and sequencing of housekeeping genes

Partial sequences of the *cts* (citrate synthase) and *rpoD* (RNA polymerase σ^70^ factor) genes of all Psy-PCR positive bacterial isolates (*n* = 197) were amplified (Table 2). In addition, a representative number of isolates (*n* = 18) were selected and sequenced with two additional housekeeping genes, *gapA* and *gyrB*. The PCR reaction mixture was the same as previously described for the Psy-PCR assay for all four housekeeping genes. PCR amplification cycles on the Bio-Rad thermal cycler (Mississauga, ON, Canada) were as follows: *cts*: 40 cycles of 30 s at 94 °C, 90 s at 62 °C, and 1 min at 72 °C; *rpoD*: 35 cycles of 1 min at 94 °C, 1 min at 57 °C, and 1 min at 72 °C; *gapA*: 30 cycles of 2 min at 94 °C, 1 min at 62 °C, and 1 min at 72 °C; *gyrB*: 35 cycles of 30 s at 94 °C, 30 s at 55 °C, and 1.5 min at 72 °C. The initial denaturation step (5 min at 94 °C) and final extension (10 min at 72 °C) were the same in the amplification cycles of all four genes. Amplicons were visualized as mentioned previously. Amplified products were sent to McLAB Laboratories for PCR purification and Sanger sequencing (McLab Laboratories, San Francisco, CA, USA). The *cts* and *rpoD* sequences obtained from McLAB were assembled using the software Geneious Prime 2022.2.2 (Boston, MA, USA). Briefly, sequences were trimmed to remove low-quality regions and consensus sequences were generated using the Geneious Assembler. The sequences were then trimmed so that all *cts* and *rpoD* sequences were the same length and were concatenated and aligned using Muscle (V. 3.8.425) implemented in Geneious Prime. Lastly, the phylogenetic tree was prepared in Geneious using PhyML (Ver. 3.3.20180621) using the maximum likelihood clustering algorithm with Jukes-Cantor (JC69) distances. A bootstrap resampling method with 1000 replicates was used to evaluate the robustness of the inferred tree. A newick file of the sequence data was imported into RStudio (version 2022.02.2) from Geneious Prime. The newick file and annotation file containing meta-data of the isolates were used in RStudio to create phylogenetic trees using ggtree. Sequences of housekeeping genes (*cts*, *gps*,* gyrB*, and *rpoD*) of 63 isolates were downloaded from National Centre for Biotechnology Information, NCBI (https://www.ncbi.nlm.nih.gov) and were used as reference isolates in the phylogenetic tree, and these represented 27 different phylogroups in the Psc (Supplementary Table S1). *Pseudomonas aeruginosa* and *Pseudomonas protegens* were used as outgroups. We also sequenced 8 Psc isolates, which were obtained from Dr. James Tambong’s lab at Agriculture and Agri-Food Canada (AAFC), Ottawa, Canada, that had previously been isolated from different hosts and characterized as diverse pathovars of *P. syringae* (Supplementary Table S1). All sequences were deposited in GenBank under accession numbers PV443409 to PV443575 for *cts* amplicons and PV468351 to PV468517 for *rpoD* amplicons.

Sequences of a subset of isolates (*n* = 16) with two additional housekeeping genes (*gyrB* and *gapA*) were used to evaluate the similarity between hierarchical clustering of the isolates in Psc between the *cts*-*rpoD* phylogenetic tree and *gyrB*-*gapA* tree. A phylogenetic tree was made with the concatenated *gyrB* and *gapA* sequences as previously described in Geneious Prime 2022.2.2 (Boston, MA, USA), and a newick file of the sequence data was imported into RStudio (version 2022.02.2). The dendrograms of the *cts*-*rpoD*, *gyrB*-*gapA*, and concatenated dendrogram of the four housekeeping genes (*cts*-*rpoD*-*gyrB*-*gapA*) trees were compared using the dendextend package in R (Galili 2015). Quantitative assessment of dendrogram similarity was conducted using Baker’s Gamma correlation coefficient (Baker 1974). This index evaluates the degree of concordance between two dendrograms by comparing the ranked heights at which each pair of elements merges in each tree. The resulting correlation ranges from − 1 (inverse structure) to + 1 (identical structure), with values near zero indicating random association. All sequences were deposited in GenBank under accession numbers PV640802 to PV640819 for *gyrB* amplicons and PV640784 to PV640801 for *gapA* amplicons.

### Genetic fingerprinting with repetitive sequence-based PCR (rep-PCR)

Genetic fingerprinting of a subset of Psy-PCR positive bacterial isolates (*n* = 116) and 8 reference isolates from Ottawa Dr. James Tambong, AAFC (Supplementary Table S1) were determined using two rep PCR assays: BOX-PCR amplifying BOXA1R primer sequences and ERIC-PCR amplifying enterobacterial repetitive intergenic consensus sequences (Table 2). PCR reactions were performed using 2.5 µl of 10X PCR buffer (New England Biolabs, Whitby, ON, Canada), 2.5 µl of 100% dimethylsulfoxide (DMSO) (Fisher Scientific, Ottawa, ON, Canada), 1 µl of 25 µM of each ERIC2 and ERIC1R primers or 2 µl of 25 µM of BOXA1R primer, 2 µl of 25 µM MgCl_2_ (New England Biolabs) for BOX-PCR or 6 µl of 25 µM MgCl_2_ (New England Biolabs) for ERIC-PCR, 3.12 µl of 10 mM dNTP (New England Biolabs), 0.4 µl of 5 U μL^−1^ of Taq polymerase (New England Biolabs, Whitby, ON, Canada), 2 µl of 20 ng µl^−1^ of genomic DNA, and the final volume was brought to 25 µl using sterile distilled water. The cycling conditions were as follows: incubation at 95 °C for 5 min followed by 35 cycles of 94 °C for 1 min, annealing for 1 min at 50 °C for primer BOXAIR and primer pair ERIC2/ERIC1R and extension at 65 °C for 4 min followed by a final extension step at 65° for 10 min. Amplified PCR fragments were run in 1.5% agarose gels (w/v) with 1× TAE buffer and SYBR safe diluted 1:10,000 (Thermo Fisher Scientific, Ottawa, Canada). The gels were visualized using a GelDoc XR+ (Bio-Rad, Mississauga, Canada), and the fragments were estimated using a 1-kb DNA Plus ladder (Invitrogen, Mississauga, Ontario, Canada).

The agarose gel pictures with amplified fragments of bacterial strains (Joint Photographic Express Group, JPEG format) were analyzed using software GelJ (SourceForge, San Diego, CA, USA) (Heras et al. 2015). The amplified bands from each well were manually selected, and band positions were normalized using a 1-kb DNA Plus ladder (Invitrogen, Mississauga, Ontario, Canada) to adjust for differences in band migration across different gels. Similarity matrices were generated using UPGMA linkage to compare all samples from both the ERIC- and BOX-PCR assays. Similarity matrices were then imported into R-Studio version 2022.02.2 (R Core Team 2022) to produce dendrograms using Provesti’s distance to compute pairwise distances based on the Provesti formula, returning a distance matrix. Prevosti’s distance was used since it is optimized for a larger number of individuals (*n* > 125). The BOX- and ERIC-PCR similarity matrices were combined to generate dendrograms in R-Studio with a cut-off of 0.015 for clustering genetically distinct groups. A neighbor joining clustering method was used to create the dendrogram.

## Results

### Disease symptoms and geographic distribution, and pathogen isolation

We monitored a total of 38 fields from 10 diverse regions of BC during 2017 to 2020 and one field from Vancouver Island in 2018 (Table 1, Fig. 1). The number of diseased fields varied across regions (Table 1). Overall, bacterial blight symptoms were observed in 88% of the total blueberry fields monitored over three years, including 100% diseased fields from five regions, the Sumas Prairie, Langley, Surrey, Delta, and Vancouver Island. In the vast majority of fields, dark brown to black necrosis in the stem and floral buds (Fig. 2A-C) was observed, although symptoms of leaf and flower necrosis were also observed in a few fields. Necrosis usually occurred from the apex of blueberry stems and progressed downward as dark, water-soaked lesions on the stems, which gradually turned black; these lesions ranged in size from a few centimeters to the entire branch. In addition, there is typically shoot dieback and blighting of the buds located near the affected area, along with withering and discoloration of flower buds and blossoms. The disease severity was higher in plants with newly planted fields (< 5 years old) than in the older blueberry orchards. A total of 380 bacterial isolates were obtained from 283 blueberry samples with blighted symptoms. Pseudomonads were not isolated from all samples, as the average frequency of pseudomonads-positive samples was 32% across all locations and varieties (Table 1). The pseudomonads-positive samples were highest in the Delta (57%) and Surrey (52%) regions (Table 1). Bacterial blight symptoms were observed in all 15 varieties monitored. The frequency of pseudomonads-positive samples also varied among the 15 varieties of blueberry. Two varieties, Draper (57%) and Aurora (50%), had the highest frequency of pseudomonads-positive samples across locations (Tables 1 and 3). All varieties except for Duke that were collected from the Surrey region had pseudomonads-positive samples, and all samples of Aurora and Draper from this region had pseudomonads-positive samples (Tables 1 and 3).

**Table 3:** Pseudomonads positive samples of major varieties of highbush blueberry grown at different locations in British Columbia

		Locations								
Variety		Abbotsford	Chilliwack	Delta	Langley	Maple Ridge	Mission	Sumas Prairie	Surrey	Total
Aurora	No. of samples	5	–^b^	–	–	–	–	5	12	**22**
	No. of pseudomonads positive samples	2 (40)^a^	–	–	–	–	–	2 (40)	7 (58)	**11 (50)**
Bluecrop	No. of samples	1	2	–	2	–	13	–	8	**26**
	No. of pseudomonads positive samples	1 (100)	1 (50)	–	0 (0)	–	1 (8)	–	4 (50)	**7 (27)**
Chandler	No. of samples	–	–	–	2	–	2	–	2	**6**
	No. of pseudomonads positive samples	–	–	–	1 (50)	–	0 (0)	–	1 (50)	**2 (33)**
Draper	No. of samples	6	6	7	3	4	–	–	4	**30**
	No. of pseudomonads positive samples	2 (33)	2 (33)	7 (100)	1 (33)	1 (25)	–	–	4 (100)	**17 (57)**
Duke^c^	No. of samples	6	10	10	–	–	12	4	1	**43**
	No. of pseudomonads positive samples	1 (17)	1 (10)	5 (50)	–	–	3 (25)	0 (0)	0 (0)	**10 (23)**
Elliott	No. of samples	1	2	–	1	–	1	–	8	**13**
	No. of pseudomonads positive samples	1 (100)	0 (0)	–	0 (0)	–	0 (0)	–	3 (38)	**5 (31)**
Liberty	No. of samples	2	–	–	3	3	–	–	2	**10**
	No. of pseudomonads positive samples	1 (50)	–	–	1 (33)	0 (0)	–	–	2 (100)	**6 (40)**
Reka	No. of samples	–	6	8	–	–	1	1	9	**25**
	No. of pseudomonads positive samples	–	1 (17)	5 (63)	–	–	0 (0)	0 (0)	4 (44)	**10 (40)**

^a^ Value in parenthesis is the percentage of pseudomonads positive samples

^b^ – = Samples were not collected in those locations

^c^ Two samples of Duke were collected from Agassiz

#### Colony morphology on selective medium

A total of 380 isolates that were fluorescent on KB medium were recovered from 283 plant samples with blighted symptoms collected between 2017 and 2020. Out of 380 fluorescent isolates on KB medium, 72% of the isolates were also fluorescent on KBC medium, a selective medium for a few specific pathovars of *P*. *syringae* including the pathovar *syringae* (Braun-Kiewnick and Sands 2001). There was variation in the frequency of fluorescent isolates on KBC among the geographic regions (Table 1). The frequency of fluorescent isolates on KBC medium was the highest in samples from Vancouver Island (100%), Agassiz (92%), and Surrey (90%), while it was the lowest in samples from Langley (29%).

### Identification of bacterial isolates with *P. syringae* specific markers

Out of 380 isolates tested with the *P. syringae* specific (Psy) markers developed by Guilbaud et al. (2016), 52% (*n *= 197) yielded a 144 bp amplicon indicating that these isolates were Psy-PCR positive (Table 1). The frequency of Psy-PCR positive isolates was 20% lower than the frequency of isolates grown in KBC, selective medium for *P*. *syringae* pv. *syringae*. The frequency of Psy-PCR positive isolates was highest from Vancouver Island (100%) and lowest from Maple Ridge (13%) and Langley (14%) (Table 1).

### Pathogenicity test

The majority of isolates (80%) were pathogenic (Table 5) and developed dark brown necrosis symptoms on blueberry leaves (Fig. 2D). Among 40 pathogenic isolates, 10, 21, and 7 were *P. avellanae*, *P. syringae*, and *P. viridiflava*, respectively (Table 5). There were no non-pathogenic isolates in the *P. viridiflava* and Phylogenomic species A groups, and the non-pathogenic counts are considerably lower for *P. syringae* (80% pathogenic and 20% non-pathogenic) and *P. avellanae* (67% pathogenic and 33% non-pathogenic).

### Biochemical characterization

A total of 11 distinct LOPAT patterns were identified among 100 isolates (Table 4). Ninety-seven isolates were from this study, and 3 reference isolates obtained from Dr. James Tambong (Ottawa, AAFC) were used for the LOPAT. The reference isolates all had a typical LOPAT result, were Psy-PCR positive and pathogenic. Among the isolates from this study, 66 had the typical pattern of L + O − P − A − T + (*Pseudomonas* group 1a), indicating these isolates were positive for levan and tobacco hypersensitivity, but negative for oxidase, pectolytic activity, and arginine dihydrolase. All of the isolates tested for LOPAT (*n* = 100) were also tested for the Psy-PCR assay. The majority of isolates (66/69, 96%) having a typical LOPAT pattern were Psy-PCR positive (Table 4). The total number of atypical LOPAT isolates was 31 and the majority of these isolates were Psy-PCR negative (22/31, 71%). A representative number of isolates were tested for pathogenicity from the typical and atypical LOPAT pattern groups (Table 4). Of the isolates tested for pathogenicity within the typical LOPAT pattern, 87% (*n* = 34) were pathogenic and 13% (*n* = 5) were non-pathogenic. Another notable atypical pattern was L + O + P − A + T + (*n* = 9), whereas the remaining atypical LOPAT patterns were found for a few isolates, ranging from 1 to 4. Among isolates with atypical LOPAT patterns tested for pathogenicity, six were pathogenic and nine were non-pathogenic (Table 4).

**Table 4:** Biochemical (levan, oxidase, pectate, arginine dihydrolase, tobacco hypersensitivity-LOPAT) results. A total of 100 isolates were tested for LOPAT with the majority of isolates having a typical (L + O − P − A − T +) LOPAT result. Of the 100 isolates 97 were from this study and 3 were reference isolates obtained from Dr. James Tambong (Ottawa, AAFC), which were used as positive control of the typical pattern (L + O − P − A − T +). These isolates were characterized with *Pseudomonas syringae* specific (Psy-PCR) marker and the pathogenicity of representative isolates was tested

LOPAT assay				Pathogenicity assay				
Pattern	No. of isolates with same pattern	Psy-PCR^+^	Psy-PCR^−^	No. of isolates	Pathogenic isolates		Non-pathogenic isolates	
					Psy-PCR^+^	Psy-PCR^−^	Psy-PCR^+^	Psy-PCR^−^
L + O − P − A − T + ^a^	69	66	3	39	34	0	5	0
L + O + P − A + T +	9	1	8	2	0	0	1	1
L + O − P − A − T −	4	4	0	2	0	0	2	0
L + O − P − A + T +	4	0	4	2	0	1	0	1
L − O − P − A − T +	3	1	2	1	0	1	0	0
L + O + P − A + T −	3	0	3	1	0	0	0	1
L − O + P − A + T −	3	0	3	1	0	0	0	1
L − O + P − A + T +	2	0	2	2	0	1	0	1
L + O + P − A − T −	1	1	0	1	1	0	0	0
L − O − P − A + T −	1	1	0	1	1	0	0	0
L − O + P − A − T −	1	1	0	1	1	0	0	0
**Total**	**100**	**75**	**25**	**53**	**37**	**3**	**8**	**5**

^a^Isolates have typical LOPAT results for *Pseudomonas syringae* group Ia

### Phylogenetic diversity with multilocus sequencing

**Fig. 4:**
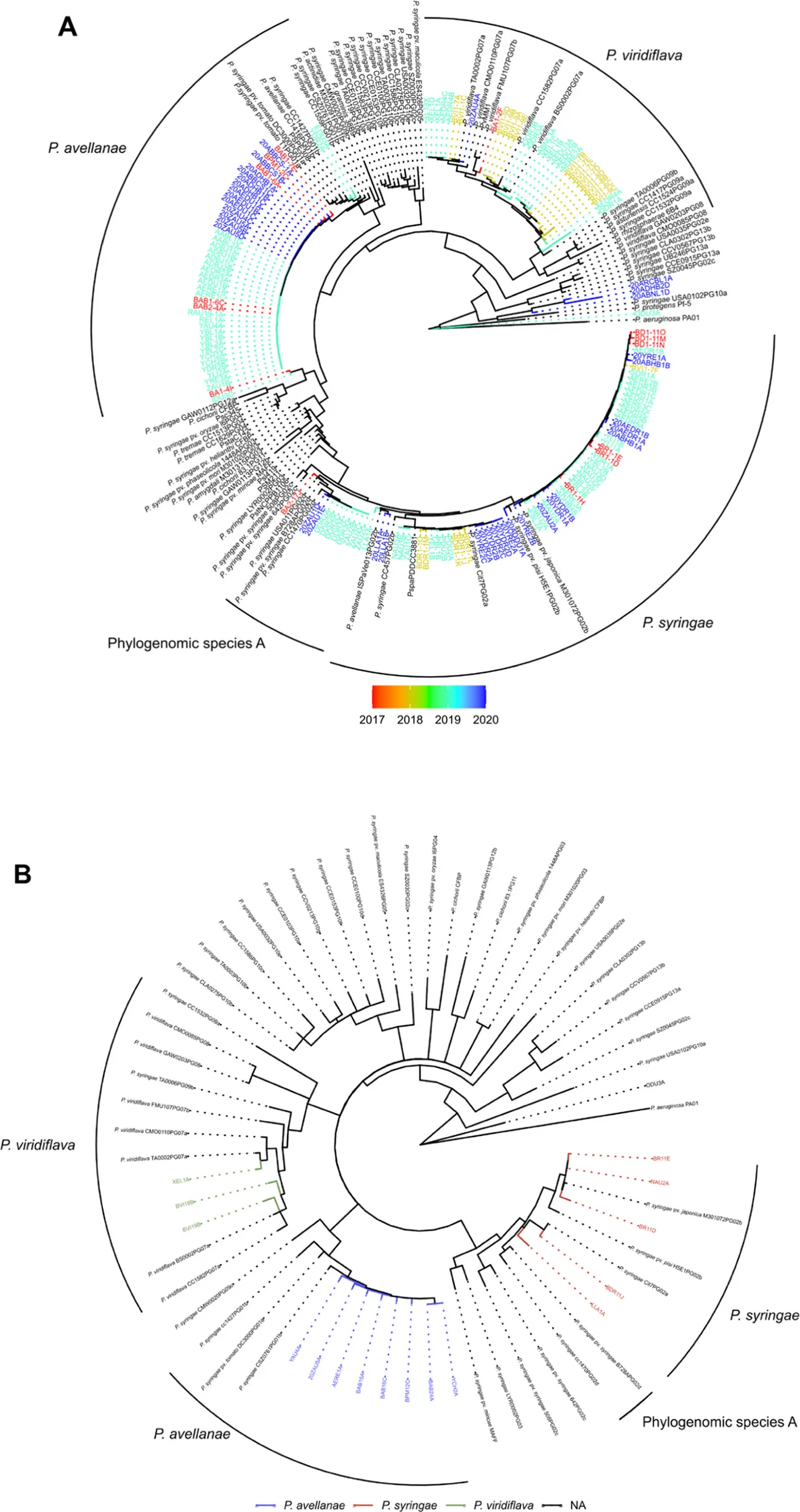
Maximum likelihood phylogenetic trees constructed using multilocus sequences (MLS) of citrate synthase (*cts*), RNA polymerase σ^70^ factor (*rpoD*) (**A**) and concatenated tree using MLS of *cts*, *rpoD*, gene encoding glyceraldehyde-3-phosphate dehydrogenase (*gapA*), and gene encoding DNA gyrase subunit B (*gyrB*) (**B**). Phylogenomic species of *Pseudomonas syringae* complex (Psc) from this study (multiple colors) were compared to reference isolates (black color, initiated with *P*., such as *P*.* syringae, P*.* virdiflava*) used by Gomila et al. (2017) to categorize the phylogenomic species within Psc. The proposed grouping of phylogenomic species is indicated by the external circle with black color. *Pseudomonas aeruginosa* and *Pseudomonas protegens* were used as outgroups

In this study, we first carried out partial sequencing of two housekeeping genes, *cts* and *rpoD*, for all Psy-PCR positive isolates (*n* = 197) to identify the phylogenomic species. Out of 197 isolates sequenced, quality and length of sequences of 43 were not good, therefore these isolates were excluded and sequences of 154 isolates were used for the phylogenetic analyses. Furthermore, a subset of representative isolates (*n* = 16) were selected and partial sequencing was performed with two additional housekeeping genes, (*gapA* and *gyrB*). Phylogenomic species of subset of 16 isolates identified using two housekeeping genes (*cts* and *rpoD*) and four housekeeping genes (*cts*, *gapA*, *gyrB*, and *rpoD*) were the same (Fig. 4). Among 154 isolates, we identified four phylogenomic species of the Psc, *Pseudomonas avellanae* (29%), *P. syringae* (40%) *Pseudomonas viridiflava* (20%), and Phylogenomic species A (7%) (Table 5; Fig. 4A). About 7% of the isolates did not belong to any known Phylogenomic species categorized by Gomila et al. (2017). Among the eight reference isolates obtained from Dr. James Tambong (AAFC, Ottawa, Canada) the isolate PspaPDDCC3881 (*P*. *syringae* pv. *papulans*) belonged to *P. syringae*, the isolate PSPE308 (*P*. *syringae* pv. *persicae*) to *P. avellanae*, the isolate PvMM1 to *P. viridiflava*, and the remaining 5 isolates were not classified into any of the four phylogenomic species of the Psc (Supplementary Table S1). Variability was observed in the frequency distribution of phylogenomic species among the years of collection geographical regions, and blueberry varieties (Table 5, Fig. 4A and Supplementary Fig. S1). Among the total isolates (*n* = 154) used in the phylogenetic studies, 54% were collected in 2019 followed by 24% in 2020. All three phylogenomic species were found in 2017, 2019, and 2020 and the frequency of *P*. *syringae* was the highest, whereas the vast majority (70%) of the isolates in 2018 were *P. viridiflava* (Table 5). About 11% and 5% of isolates in 2019 and 2020 were phylogenomic species A, respectively (Table 5). Isolates of *P*. *syringae* and *P. avellanae* were found in fields of 9 and 7 geographical regions, respectively; whereas isolates of *P. viridiflava* were only found in fields of 5 geographical regions. In particular, 93% of isolates collected from Vancouver Island (variety Draper) were *P. viridiflava*.

**Table 5:** Summary of phylogenetic tree of concatenated *rpoD* and *cts* gene sequences represented in Fig. 6. The phylogenomic species, *P. avellanae*, *P. syringae*, *P. viridiflava*, and Phylogenomic species A, classification system used by Gomila et al. (2017) were used to highlight the species diversity of *P. syringae* isolates from blueberry plants in the Lower Mainland based on of the year and location of sample collection the isolate, variety of blueberry the sample was collected from, the pathogenicity and blast match of the isolate

		% frequency of				
	No. of isolates	NC^a^	*P. avellanae*	*P. syringae*	*P. viridiflava*	Phylogenomic species A
Year						
2017	14	7	43	43	7	0
2018	20	0	0	30	70	0
2019	83	2	31	37	18	11
2020	37	8	35	49	3	5
No. of isolates	154	6	45	61	31	11
% frequency		4	29	40	20	7
Location						
Abbotsford	13	8	46	23	23	0
Agassiz	4	50	25	0	25	0
Chilliwack	12	0	8	50	0	42
Delta	46	4	39	41	15	0
Langley	1	0	0	100	0	0
Lindell Beach	1	0	0	100	0	0
Maple Ridge	1	0	100	0	0	0
Mission	6	0	17	83	0	0
Sumas Prairie	9	0	0	100	0	0
Surrey	46	0	39	37	13	11
Vancouver Island	15	0	0	7	93	0
No. of isolates	154	5	46	62	31	10
% frequency		3	30	40	20	6
Variety						
Aurora	22	0	32	41	5	23
BC Variety	3	0	100	0	0	0
Bluecrop	17	6	35	59	0	0
Bluejay	4	0	100	0	0	0
Chandler	2	0	100	0	0	0
Draper	50	0	8	42	40	10
Duke	13	15	46	23	15	0
Elliott	7	0	14	14	71	0
Hardy Blue	7	14	43	43	0	0
Lastcall	3	0	0	100	0	0
Liberty	3	0	33	67	0	0
Northland	2	50	0	0	50	0
Reka	14	0	21	71	7	0
Unknown	7	0	86	0	14	0
No. of isolates	154	5	46	62	31	10
% frequency		3	30	40	20	7
Pathogenicity						
N^b^	50	2	15	25	7	1
Pathogenic	40 (80)^c^	1 (50)	10 (67)	21 (84)	7 (100)	1 (100)
Non-pathogenic	10 (20)	1 (50)	5 (33)	4 (16)	0 (0)	0 (0)

^a^ *NC* not clustered in any of the 4 phylospecies

^b^ Total number of isolates tested for pathogenicity

^c^ Value in parenthesis is the percentage of isolates that were pathogenic or non-pathogenic

Isolates belonging to phylogenomic species A were only detected in fields from Chilliwack and Surrey. Frequency distribution of four phylogenomic species among isolates collected from 13 varieties of highbush blueberry are summarized in Table 5. Among 13 varieties, Draper had the highest number of isolates (32%), followed by Aurora (14%), Bluecrop (11%), Reka (9%), and Duke (8%). Interestingly, three phylogenomic species *P. avellanae*, *P*. *syringae*, and *P. viridiflava* were detected from these four varieties except Bluecrop. *P*. *avellanae* and *P*. *syringae* were present in most of the varieties, while *P. viridiflava* was present in six varieties, and Phylogenomic species A was only present in Auroa and Draper. Chandler, Bluejay, and BC varieties had exclusively isolates of *P. avellanae* that could be due to small numbers of isolates collected from these varieties.

### Genetic fingerprinting with rep-PCR

**Fig. 5:**
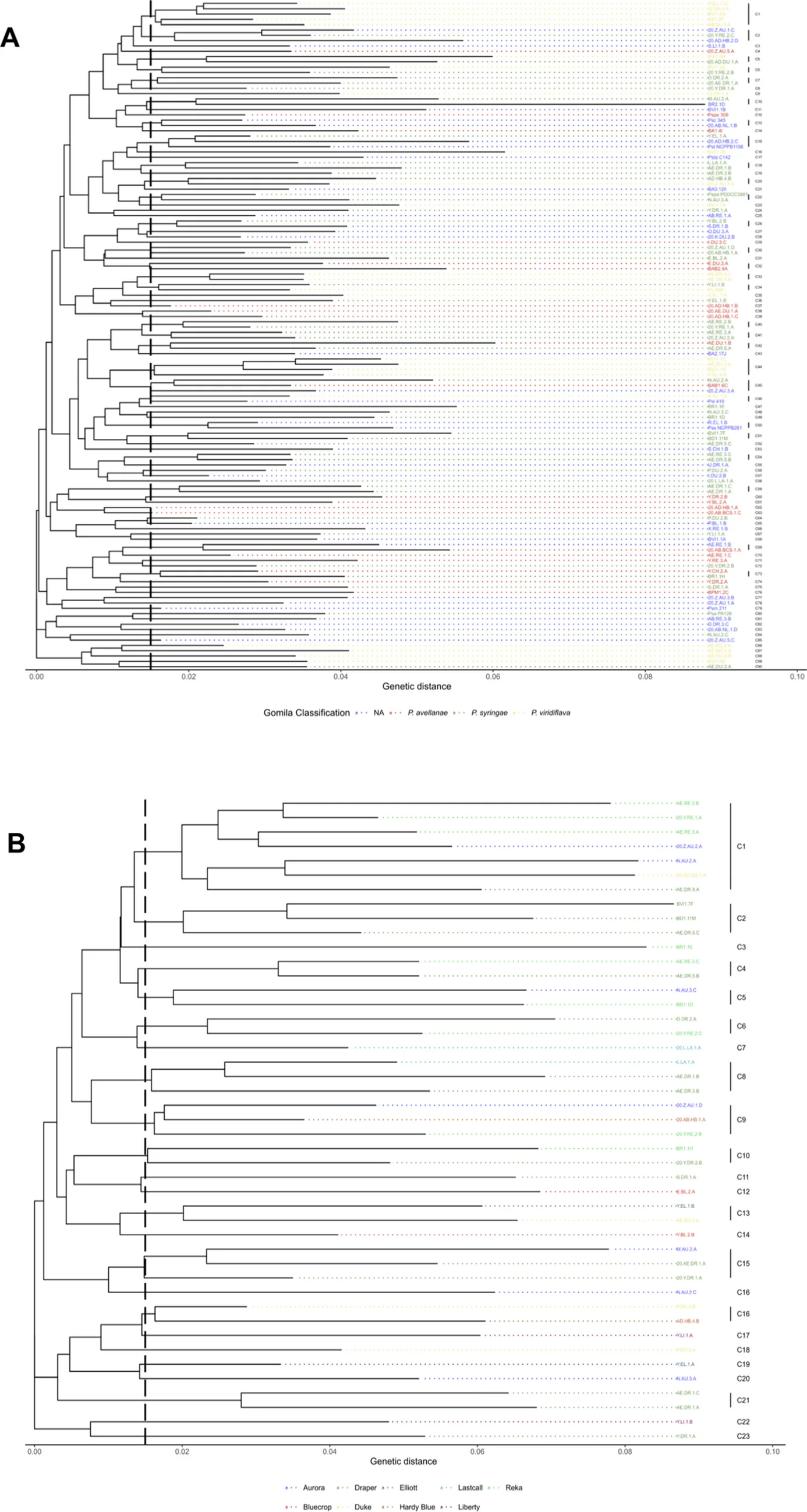

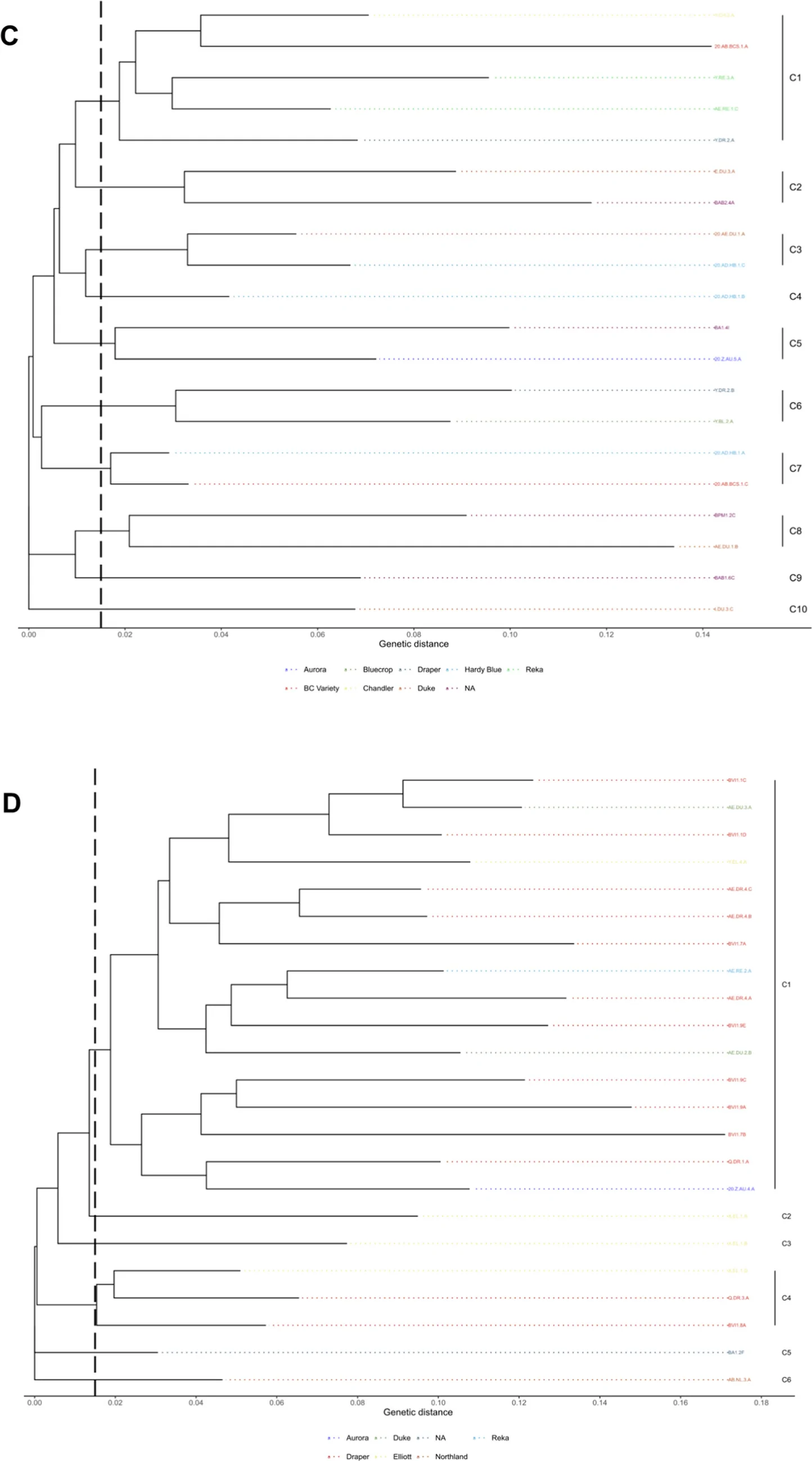
Dendrograms of isolates of *Pseudomonas syringae* complex (Psc) from highbush blueberry, which were generated after the cluster analyses of combined rep-PCR fingerprints using ERIC- and BOX-PCR assays. Clusters were determined to be at a genetic distance of 0.015. **A** Psc isolates of all phylogenomic species (*P. syringae*, *P. avellanae*, *P. viridiflava*) based on the classification of Gomila et al. (2017), isolates of each phylogenomic species have different colors (**B**–**D**). Psc isolates of each phylogenomic species colored based on blueberry varieties: *P. syringae* (**B**), *P. avellanae* (**C**), *P. viridiflava* (**D**). NA indicates phylogenomic species were not detected

BOX- and ERIC-PCR assays had 99 and 86 unique loci respectively ranging from 75 to 3000 bp (Supplementary Figs. S2 and S3). There were 126 unique patterns with 90 clusters in the combined assays on BOX- and ERIC-PCR for all three phylogenomic species (Fig. 5A). Of the 90 clusters found among the isolates, 28 clusters had more than one isolate (Fig. 5A). Of the 28 clusters that had more than one isolate, there were 15 clusters that had isolates of the same phylospecies. Fingerprinting analysis using the two rep-PCR assays showed a high genetic variation among the blueberry isolates in BC, but these assays were not able to discriminate phylogenomic species of Psc (Fig. 5A). The rep-PCR fingerprinting was also used to create a dendrogram among isolates from different blueberry varieties within each phylogenomic species (Fig. 5B–D). 5B-D Isolates of *P. syringae* had the highest numbers (23) of clusters (Fig. 5B), isolates of *P. avellanae* had 10 clusters (Fig. 5C), and isolates of *P. viridiflava* had the lowest (6) clusters (Fig. 5D).

**Fig. 6:**
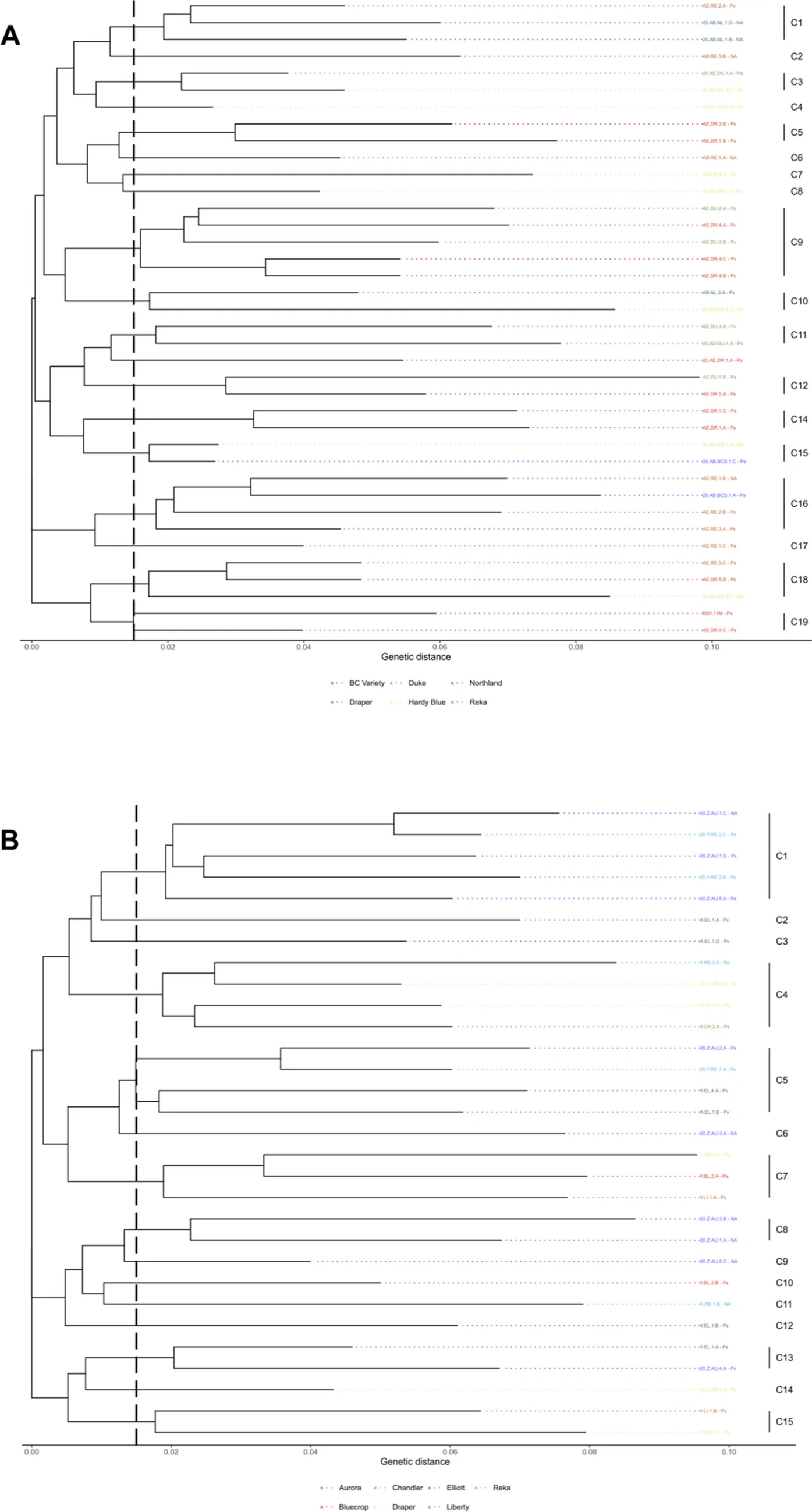

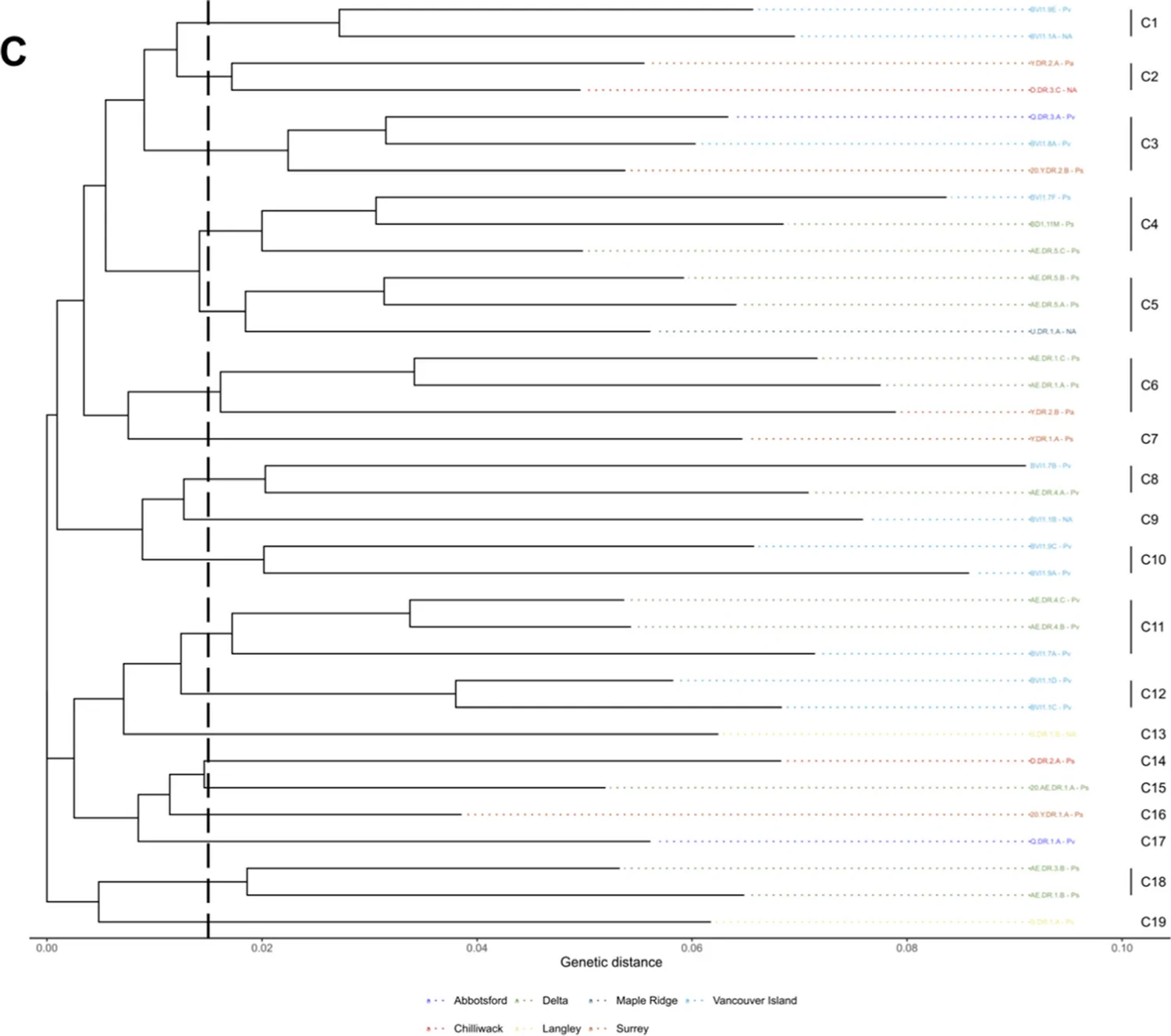
Dendrograms of isolates of *Pseudomonas syringae* complex (Psc) grouped based on highbush blueberry varieties and geographic locations. Dendrograms were generated after the cluster analyses of combined rep-PCR fingerprints using ERIC- and BOX-PCR assays. Clusters were determined to be at a genetic distance of 0.015. Psc isolates colored by blueberry variety were collected from the geographic locations Delta (A) and Surrey (B), and Psc isolates colored by geographic locations were isolated from the variety Draper (C). The last two letters of the pathogen isolate code indicate the phylogenomic species (Ps = *P. syringae*, Pa = *P. avellanae*, Pv = *P. viridiflava*, and NA = Not classified)

To evaluate the impact of varieties in genetic clustering of isolates at specific locations, dendrograms of isolates from two geographical regions, Delta (*n* = 38) and Surrey (*n* = 30), were constructed separately (Fig. 6A and B). The isolates had a high genetic diversity as indicated by the high number of clusters in the two locations, and the Draper variety. There was some impact of locations and varieties, but no distinct pattern was observed. At the Delta location, there was a total of 19 clusters, with 12 clusters having more than one isolate (Fig. 6A). Of these 12 clusters, isolates from the same blueberry variety grouped in each of 7 clusters, while 8 clusters had isolates of the same phylogenomic species in each cluster. At the Surrey location, there were a total of 15 clusters, with 7 clusters having more than one isolate (Fig. 6B). Of these 7 clusters, there were 4 clusters from the same blueberry variety and 3 that had isolates of the same phylogenomic species. In addition, a dendrogram among Psc isolates from the Draper blueberry variety (*n* = 35) was constructed to determine if there was clustering among isolates of the same variety from different geographic locations (Fig. 6C). There was a total of 19 clusters, each of which had 11 clusters having more than one isolate. Of these 11 clusters, there were 6 that had isolates from the same location and 8 that had isolates of the same phylogenomic species (Fig. 6C).

## Discussion

This is the first comprehensive study in the highbush blueberry production region of Canada, monitoring the prevalence of bacterial blight and canker from multiple years, diverse geographical areas, and varieties. Using microbiological, biochemical, and molecular tools, we further characterized a large collection of pseudomonads (*n* = 380) isolated from 283 blueberry samples (15 varieties) collected from 36 commercial farms and two research farms located in ten diverse geographical areas. Bacterial blight symptoms were found in all varieties monitored between 2017 and 2020; however, the disease symptoms were severe and the frequency of *Pseudomonas* isolation from diseased samples was higher in varieties Draper and Aurora than in other varieties surveyed, indicating that these varieties are susceptible to the disease. We also found bacterial blight in 88% of the total monitored fields, confirming that the disease is endemic and widespread across BC and Vancouver Island and that the environmental conditions (high rainfall in the spring and fall, mild temperature, and high humidity in early summer) in the region create favorable conditions for pathogen proliferation and infection to blueberry plants (Anonymous 2025). Compared to other geographical areas, higher levels of pathogen infection (as indicated by pseudomonads positive disease samples) were found on commercial farms of Delta (57%) and Surrey (52%); this variability could be due to differences in microclimates (frost occurrences) and agricultural practices across the surveyed regions (Vanneste et al. 2015; Lahlali et al. 2024). In BC, the occurrence of bacterial blight in highbush blueberry was also reported previously (Canfield et al. 1986; Joshi 2003; Joshi and Jeffries 2010), about 33% of isolates from blueberry were positive (Canfield et al. 1986). The 1986 disease was also reported as endemic with sporadic diseases of highbush blueberry in the Pacific Northwest region of the USA (Stockwell et al. 2015), Poland (Kałużna et al. 2013), and Serbia (Zlatković et al. 2022). The economic impact of the bacterial blight and canker caused by species of the Psc in other fruit crops is well documented. For example, bacterial canker caused by *P. syringae* pv. *actinidiae* in kiwifruit affected 37% of New Zealand’s kiwifruit orchards and over 70% of the area planted in kiwifruit experiencing damage, resulting in an industry cost exceeding $1.3 billion (Frampton et al. 2014; Vanneste 2017). In Germany, bacterial canker of plum leads to annual tree mortality rates as high as 30% (Hinrichs-Berger 2004), and winter pruning of plum trees resulted in mortality rates between 57 and 100% in Georgia, USA due to virulent *P. syringae* isolates (Chandler and Daniell 1976). Future research is required to quantify the economic impact of bacterial blight on highbush blueberry in Canada.

Using species-specific PCR assay (Psy) developed by Guilbaud et al. (2016), we found that 52% of the isolates were Psy-PCR positive. Guilbaud et al. (2016) reported that the Psy assay can identify isolates of the Psc; however, the assay can’t discriminate phylogenomic species (Berge et al. 2014). Multilocus sequencing with housekeeping genes in our study identified four distinct phylogenomic species within the Psc; isolates of *P. syringae* (40%) were most prevalent, followed by *P. avellanae* (29%), *P. viridiflava* (20%), and Phylogenomic species A (7%). To our knowledge, this is the first report of three phylogenomic species, *P. avellanae*, *P. viridiflava,* and Phylogenomic species A in highbush blueberry. We also found variability in the frequency distribution of phylogenomic species of the Psc among geographic areas and blueberry varieties. For example, isolates of *P. avellanae* and *P. syringae* were found in about two-thirds or higher of the geographical areas of BC, while the majority of isolates of *P*. *viridiflava* were found from the field of Vancouver Island. About 70% of isolates of *P*. *viridiflava* were recovered from the variety Draper, while a few isolates were recovered from the other five varieties. Previous studies have documented the prevalence of *P. syringae* as a major pathogen in temperate fruit crops due to its wide host range and adaptability to environmental conditions (Lamichhane et al. 2015; Morris et al. 2013).

*P. syringae* pv. *syringae* was reported as the causal agent of bacterial blight and canker of highbush blueberry in Canada (Canfield et al. 1986; Joshi 2003; Joshi and Jeffries 2010); however, identification of the pathogen was mainly based on phenotypic characteristics. The designation of pathovars of *P. syringae* was based on the hosts from which the pathogen was isolated and the specific pathogenicity towards a potential host (Bull et al. 2010). However, the pathovar *syringae* can infect more than 40 plant species (Young 1991), and the host specificity of the pathogen may not justify the formal differentiation among species (Young 2010). Based on phenotypic similarities, taxonomically closely related species were considered as a Psc (Young 2010). Using multi-locus sequencing and whole genome sequencing, Gomila et al. (2017) reclassified and identified 14 phylogenetic species within a Psc. Their studies included 15 pathovars in the phylogenetic analyses, and pathovars are aligned with the phylogenomic species, as in our phylogenetic analyses. Among these, three significant phylogenetic species of the *P. syringae* species complex were identified in this study (*P. syringae*, *P. avellanae*, and *P. viridiflava*).

All three phylogenetic species have evolved to infect a wide range of economically significant crops, causing substantial agricultural losses. *P. syringae* pv*. syringae* is a known pathogen of cherry trees, with *P. syringae* pv*. syringae* exhibiting greater aggressiveness in sweet cherries (Latorre and Jones 1979; Ilicic et al. 2018). In addition, *P. syringae* pv*. syringae* has been identified as the causal agent in citrus blast (Ivanović et al. 2017; Oueslati et al. 2022) and bacterial inflorescence rot in grape (Hall et al. 2016; Whitelaw-Weckert et al. 2011). Similarly, *P. syringae* pv*. syringae* is a major limiting factor in global mango production due to bacterial apical necrosis (Gutierrez-Barranquero et al. 2012), and cucurbit crops causing leaf spot and fruit warts (Tymon and Inglis 2017). *Pseudomonas syringae* pv*. actinidiae*, also classified as *P. avellanae* by Gomila et al. (2017), has emerged as a pandemic threat to kiwifruit cultivation worldwide, leading to severe symptoms such as leaf necrosis, flower blight, and substantial reductions in fruit yield (Cameron and Sarojini 2014; Pinheiro et al. 2020).

*P*. *viridiflava* represents another significant phytopathogen with a broad host range. In recent years, atypical *P. viridiflava* strains caused infections in multiple crops. In Chile, *P. viridiflava* was first identified as the causal agent of fruit rot in sweet cherry trees, demonstrating copper resistance and carrying pathogenicity-related genes such as *avrE* and *hrpL* (Beltrán et al. 2023). In southern Italy, the bacterium was linked to bacterial diseases in Molfettese Catalogna chicory, sugarloaf chicory, curly lettuce, and safflower, where it caused leaf necrosis, streaking, and plant death (Cariddi et al. 2025)​. Additionally, in Greece, *P. viridiflava* was reported as a new pathogen in common and spiny chicory, leading to severe leaf blight and crop losses of up to 30% (Trantas et al. 2022).

LOPAT biochemical tests have typically been used to identify *P. syringae* from other fluorescent pseudomonads (e.g., *P. viridiflava*). About 69% of Psc isolates had the typical LOPAT pattern (Table 4). However, the rest of the isolates displayed atypical LOPAT patterns that have been observed in other hosts such as bean, kiwifruits, cucumber, and lettuce (González et al. 2003; Olczak-Woltman et al. 2007). The majority of isolates tested had the typical results for *P. syringae* and contained all four phylogenomic species present in the phylogenetic tree. These results are typical for *P. syringae*, phylogenomic species A, and *P. avellanae*, and all three fall into group Ia of the LOPAT determinative scheme. However, *P. viridiflava*, which generally has L − O − P + A − T + (group II) results, was also shown to have the same LOPAT results as group Ia. Atypical LOPAT results have not been observed in BC for *P. viridiflava*, but González et al. (2003) have previously established atypical LOPAT results (group Ia) in bean plants from Spain.

The genetic diversity observed in the combined rep-PCR assays reflects the complex population structure of *P. syringae* in BC. The 28 clusters that contained more than one isolate and the presence of isolates from multiple years, locations, and blueberry varieties within these clusters suggest that the pathogen is widely disseminated and has adapted to diverse environmental conditions and host varieties (Gomila et al. 2017). Interestingly, despite this widespread distribution, no distinct genetic clustering was observed when isolates were analyzed by phylospecies, year, location, or blueberry variety, suggesting that there may be significant genetic overlap and gene flow among *P. syringae* populations across the region. This lack of clear geographic or host-specific differentiation is in line with findings from previous studies on *P. syringae*, where high genetic variability is often seen regardless of environmental factors or host specificity (Sarkar and Guttman 2004; Nikolić et al. 2018; Peng et al. 2022). Given this initial lack of distinct clustering, the analysis was narrowed to focus on two specific locations, Delta and Surrey, which had a high number of isolates, as well as the Draper blueberry variety. This approach aimed to reduce the inherent variability in the fingerprinting assay and detect potential location- or variety-specific patterns in the population structure of *P. syringae*. In Delta, a total of 22 clusters were identified, with 12 clusters containing more than one isolate. Of these, four clusters contained isolates from the same blueberry variety, and six clusters were composed of isolates from the same phylogenomic species. These findings suggest some degree of localized genetic clustering within Delta, potentially influenced by the specific agricultural practices or microclimatic conditions at this location (Sundin et al. 2016). The presence of isolates from the same phylogroup within these clusters points to the persistence of certain *P. syringae* lineages within the Delta location, possibly due to factors such as continuous host availability or environmental stability that allow for the survival and spread of particular strains (Hirano and Upper 2000; González et al. 2003). In Surrey, 15 clusters were identified, and while fewer clusters were observed in Surrey compared to Delta, the trend of phylogroup-based clustering remained consistent. This suggests that certain *P. syringae* lineages may be more prevalent or successful within specific locations, possibly due to location-specific environmental or agricultural factors that favor the proliferation of particular strains (Loper et al. 2012). A total of 19 clusters were identified for the Draper variety across different locations, with 11 clusters containing more than one isolate. The prevalence of phylogroup-based clustering in Draper is notable, as it may indicate the presence of host-specific adaptations that allow certain *P. syringae* strains to more effectively colonize this variety (Sarkar and Guttman 2004; Choi et al. 2017).

Understanding the identity of bacterial plant pathogens holds paramount importance in establishing effective and sustainable disease management strategies within agricultural systems. This knowledge carries significant weight for growers, diagnosticians, extension agents, and other stakeholders engaged in crop cultivation. However, such identifications are intricately tied to the complex and ever-evolving realm of bacterial taxonomy, a discipline that relies on methods and data often not commonly employed by those engaged in field issue diagnosis. Additionally, the immune responses of younger plants might not be as robust or fully developed as in mature plants, rendering them more vulnerable to rapid bacterial colonization and subsequent symptom expression. In contrast, older blueberry plants typically exhibit a higher degree of resistance to infection; however, older plants can still serve as reservoirs for the pathogen, perpetuating the disease cycle within blueberry plantations. Comprehensive characterization is crucial for developing targeted breeding programs aimed at enhancing genetic resistance in blueberry plants, ultimately mitigating the impact of bacterial blight in the region. The potency of molecular tools in precisely pinpointing bacterial pathogenicity extends its significance to a diverse audience, ranging from farmers and diagnosticians to phytobacteriologists, and taxonomists.

## Supplementary information

Below is the link to the electronic supplementary material.

ESM 1 (PDF 1.03 MB)

## Data Availability

All data generated or analyzed during the study is included in this published article and its supplementary information.
