# Supplementary material for: Characterization and genetic diversity of pseudomonads population from highbush blueberry in western Canada
Source: Appl Microbiol Biotechnol. 2026 Jan 12;110(1):15. doi: 10.1007/s00253-025-13676-y (PMC12795873; doi:10.1007/s00253-025-13676-y)
Supplement: Supplementary file 1 — (PDF 1.03 MB) [file 253_2025_13676_MOESM1_ESM.pdf]

Journal: Applied Microbiology and Biotechnology

Supplemental Materials

Characterization and genetic diversity of pseudomonads population from highbush blueberry in western Canada

Someshwar R. Latchman[1], Rishi R. Burlakoti[1†], Amy Novinscak[1], Simone D. Castellarin[2]

[1]Agassiz Research and Development Centre, Agriculture and Agri-Food Canada, 6947 Hwy 7, Agassiz, BC, V0M 1A0, Canada

[2]Wine Research Centre, Faculty of Land and Food Systems, The University of British Columbia, Vancouver, BC, Canada

[†]Corresponding author: Rishi R. Burlakoti
Email: rishi.burlakoti@agr.gc.ca; Phone:604-316-0501; Fax: 604-796-6133

Table S1: Reference isolates used in the phylogenetic tree that represented 27 different phylogroups in the *Pseudomonas syringae* complex. First 63 isolates were downloaded from NCBI and 8 isolates* were obtained from Dr. James Tambong's lab at Agriculture and Agri-Food Canada (AAFC), Ottawa, Canada.

SN	Isolate code	Phylogroup	Gomila et al. 2017 Classification
1	*Pamygdali*M301315PG03	3	*P. amygdali*
2	*Psyringae*LYR0002PG03	3	*P. amygdali*
3	*Psyringaemiricae*MAFF	3	*P. amygdali*
4	*Psyringaemori*M301020PG03	3	*P. amygdali*
5	*Psyringaephaseolicola*1448APG03	3	*P. amygdali*
6	*Psyringaeoryzae*I6PG04	4	*P. coronafaciens*
7	*Ptremae*CC1513PG04	4	*P. coronafaciens*
8	*Ptremae*CC1629PG04	4	*P. coronafaciens*
9	*Psyringaemaculicola*ES4326PG05	5	*P. cannabina*
10	*Psyringaehelianthi*CFBP	6	*P. caricapapayae*
11	*Pviridiflava*CMO0085PG08	8	*P. viridiflava*
12	*Pviridiflava*GAW0203PG08	8	*P. viridiflava*
13	*Pcichorii*83.1PG11	11	*P. cichorii*
14	*Pcichorii*CFBP	11	*P. cichorii*
15	*Psyringae*CC1583PG10a	10a	Phylogenomic species B
16	*Psyringae*CCE0103PG10a	10a	Phylogenomic species B
17	*Psyringae*USA0102PG10a	10a	Phylogenomic species B
18	*Psyringae*CLA0275PG10b	10b	Phylogenomic species B
19	*Psyringae*TA0003PG10b	10b	Phylogenomic species B
20	*Psyringae*TA0019PG10b	10b	Phylogenomic species B
21	*Psyringae*CC1586PG10c	10c	Phylogenomic species B
22	*Psyringae*CCE0100PG10d	10d	Phylogenomic species B
23	*Psyringae*USA0032PG10e	10e	Phylogenomic species B

	Isolate Code	Phylogroup	Gomila et al. 2017 Classification
24	*Psyringae*CCE0153PG10f	10f	Phylogenomic species B
25	*Psyringae*CCV0213PG10g	10g	Phylogenomic species B
26	*Psyringae*GAW0112PG12a	12a	N.A
27	*Psyringae*GAW0113PG12b	12b	N.A
28	*Psyringae*CCE0915PG13a	13a	Phylogenomic species D
29	*Psyringae*UB246PG13a	13a	Phylogenomic species D
30	*Psyringae*CCV0567PG13b	13b	Phylogenomic species D
31	*Psyringae*CLA0302PG13b	13b	Phylogenomic species D
32	*Psyringae*tomatoDC3000PG01a	1a	*P. tomato*
33	*Psyringae*tomatoT1PG01a	1a	*P. tomato*
34	*Pactinidiae*M302091PG01b	1b	*P. avellanae*
35	*Pavellanae*CC1416PG01b	1b	*P. avellanae*
36	*Psyringaecc*1427PG01b	1b	*P. avellanae*
37	*Psyringae*CC1559PG01b	1b	*P. avellanae*
38	*Psyringae*CSZ0761PG01b	1b	*P. avellanae*
39	*Psyringae*Cit7PG02a	2a	N.A
40	*Pavellanae*ISPaVe013PG02b	2b	*P. syringae*
41	*Psyringae*CC457PG02b	2b	*P. syringae*
42	*Psyringaejaponica*M301072PG02b	2b	*P. syringae*
43	*Psyringaepisi*H5E1PG02b	2b	*P. syringae*
44	*Psyringaesyringae*508PG02c	2c	*P. congelans*
45	*Psyringaesyringae*642PG02c	2c	*P. congelans*
46	*Psyringae*SZ0030PG02c	2c	*P. congelans*
47	*Psyringae*SZ0045PG02c	2c	*P. congelans*
48	*Psyringaecc*1470PG02d	2d	Phylogenomic species A
49	*Psyringaesyringae*B728APG02d	2d	Phylogenomic species A
50	*Psyringae*USA011PG02d	2d	Phylogenomic species A
51	*Psyringae*USA0035PG02e	2e	N.A
52	*Pviridiflava*BS0002PG07a	7a	*P. viridiflava*
53	*Pviridiflava*CC1582PG07a	7a	*P. viridiflava*
54	*Pviridiflava*CMO0110PG07a	7a	*P. viridiflava*
55	*Pviridiflava*TA0002PG07a	7a	*P. viridiflava*
56	*Pviridiflava*FMU107PG07b	7b	*P. viridiflava*
57	*Pasturiensis*CC1524PG09a	9a	Phylogenomic species C
58	*Psyringae*CC1417PG09a	9a	Phylogenomic species C
59	*Psyringae*CC1532PG09a	9a	Phylogenomic species C
60	*Psyringae*TA0006PG09b	9b	Phylogenomic species C
61	*Psyringae*CMW0020PG09c	9c	Phylogenomic species C
62	*Paeruginosa*PA01	*P. aeruginosa*	
63	*Pprotegens*Pf-5	*P. protegens*	
64	Psc345* (*P. coronafaciens*)	N.A	N.A

	Isolate Code	Phylogroup	Gomila et al. 2017 Classification
65	Psl419* (*P. syringae* pv. *lachrymans*)	N.A	N.A
66	PSM211* (*P. syringae* pv. *morsprunorum)*	N.A	N.A
67	PspaPDDCC3881*(*P. syringae* pv. *papulans*)	2b	*P. syringae*
68	PSPE308*(*P. syringae* pv. *persicae*)	1b	*P. avellanae*
69	PstNCPPB*1106 (*P. syringae* pv. *tomato*)	N.A	N.A
70	PstaC142*(*P. syringae* pv. tagetis)	N.A	N.A
71	PvMM1*(*P. viridiflava*)	7a	*P. viridiflava*

N.A = Not aligned to phylogenomic species

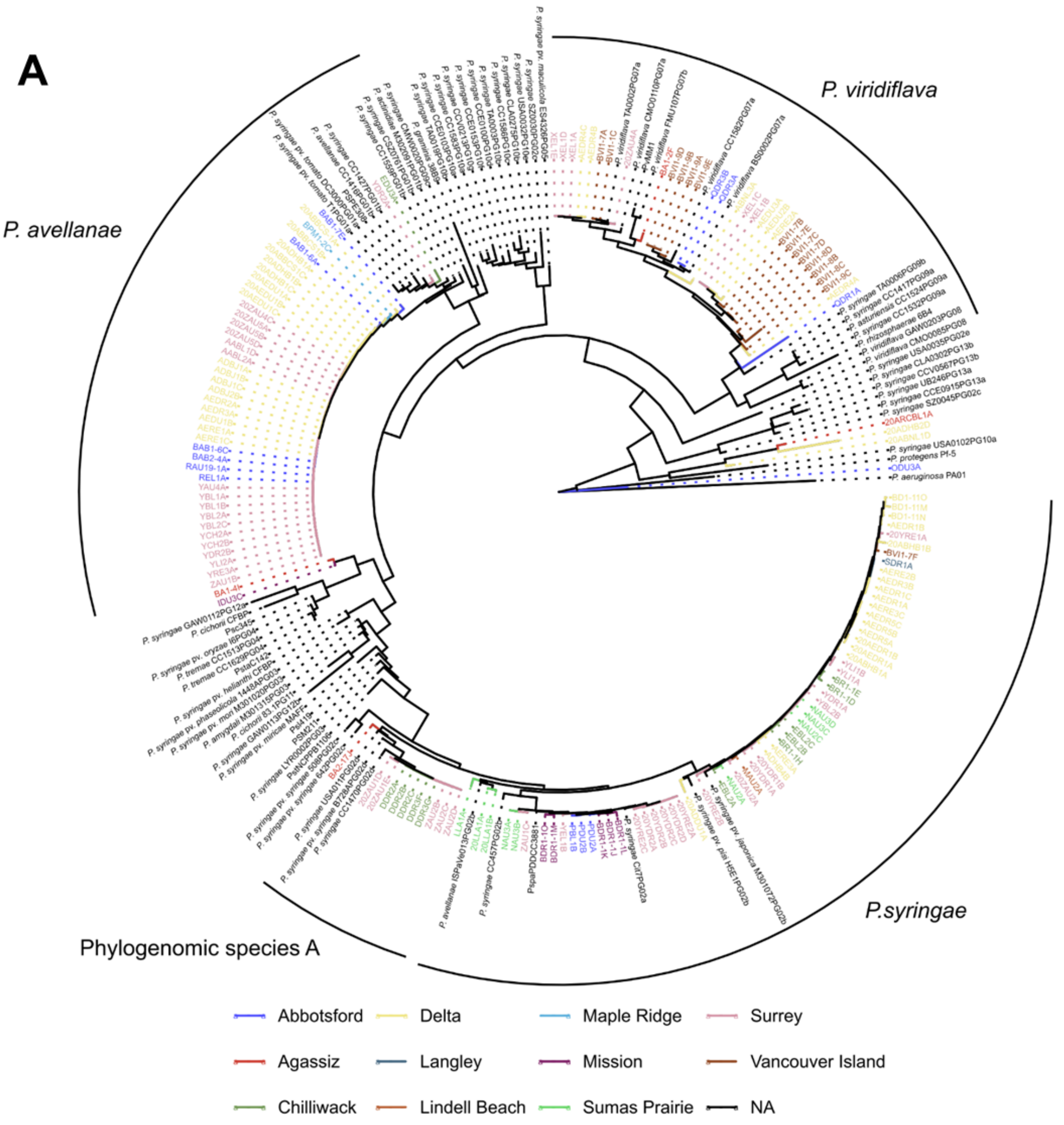

A
P. viridiflava
P. avellanae
Phylogenomic species A
P.syringae
Abbotsford
Delta
Maple Ridge
Surrey
Agassiz
Langley
Mission
Vancouver Island
Chilliwack
Lindell Beach
Sumas Prairie
NA

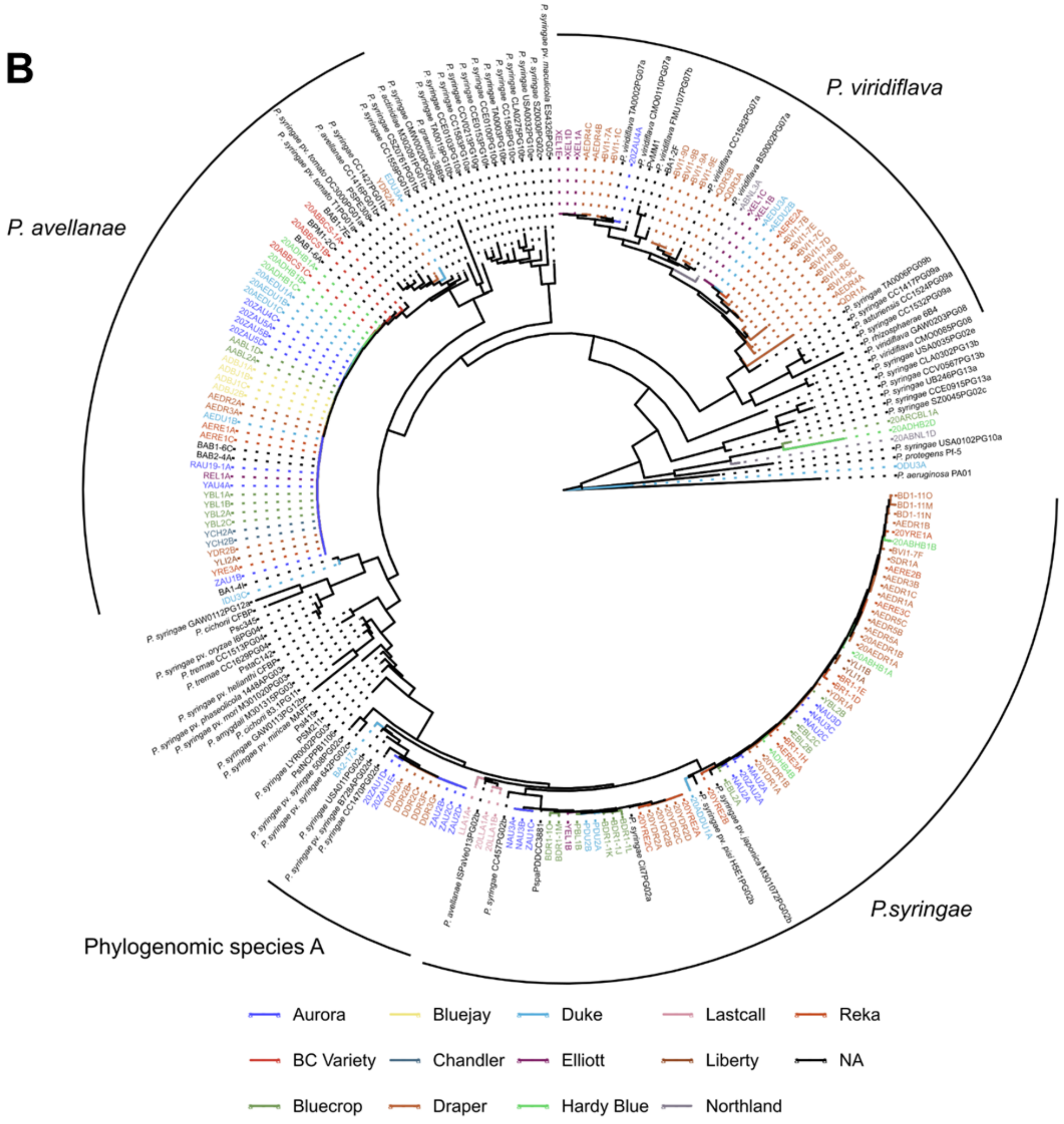

Fig. S1 Maximum likelihood phylogenetic trees constructed using multilocus sequences (MLS) of citrate synthase (*cts*), RNA polymerase σ^{70} factor (*rpoD*). Phylogenomic species of *Pseudomonas syringae* complex (Psc) from this study (multiple colours) were compared reference isolates (black colour, initiated with *P*., such as *P syringae, P virdiflava*) used by Gomila et al. (2017) to categorize the phylogenomic species within Psc. The proposed grouping of phylogenomic species are indicated by the external circle with black colour. *Pseudomonas aeruginosa*, and *Pseudomonas protegens* were used as outgroups. The phylogenetic trees show the species diversity of Psc strains isolated from highbush blueberry plants from British Columbia based on the geographic location (A), variety of highbush blueberry (B).

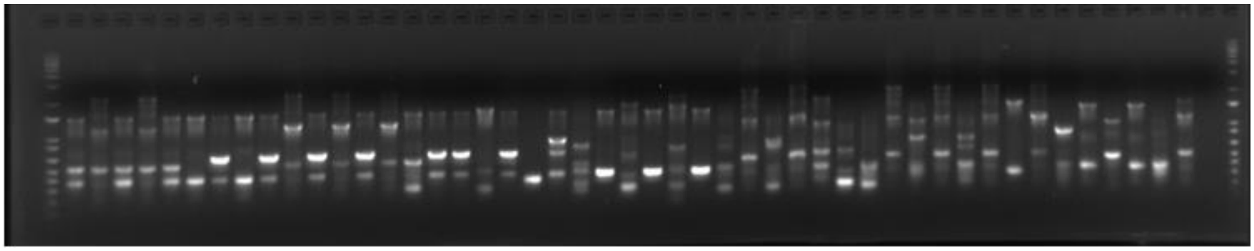

Fig. S2 Example of gel picture showing DNA fingerprints of isolates of *Pseudomonas syringae* complex amplified with BOX-PCR assy.

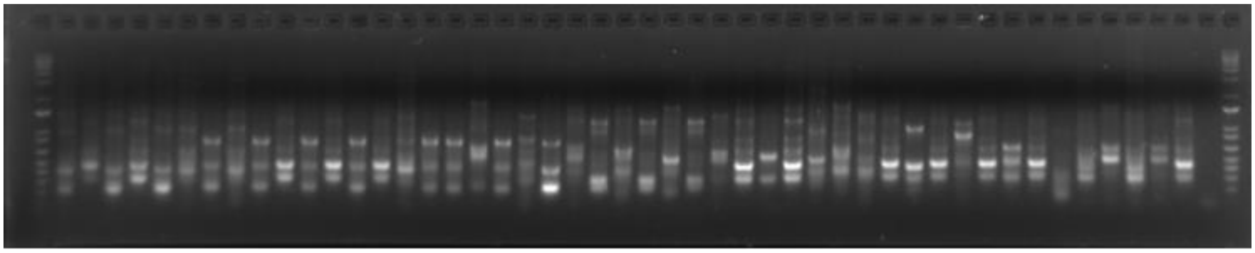

Fig. S3 Example of gel picture showing DNA fingerprints of isolates of *Pseudomonas syringae* complex amplified with ERIC PRC assy.
